# Fast Bayesian inference in large Gaussian graphical models

**DOI:** 10.1111/biom.13064

**Published:** 2019-05-06

**Authors:** Gwenaël G. R. Leday, Sylvia Richardson

**Affiliations:** ^1^ MRC Biostatistics Unit School of Clinical Medicine, University of Cambridge Cambridge UK

**Keywords:** Bayes factor, correlation, Gaussian graphical model, high‐dimensional data, inverse‐Wishart distribution

## Abstract

Despite major methodological developments, Bayesian inference in Gaussian graphical models remains challenging in high dimension due to the tremendous size of the model space. This article proposes a method to infer the marginal and conditional independence structures between variables by multiple testing, which bypasses the exploration of the model space. Specifically, we introduce closed‐form Bayes factors under the Gaussian conjugate model to evaluate the null hypotheses of marginal and conditional independence between variables. Their computation for all pairs of variables is shown to be extremely efficient, thereby allowing us to address large problems with thousands of nodes as required by modern applications. Moreover, we derive exact tail probabilities from the null distributions of the Bayes factors. These allow the use of any multiplicity correction procedure to control error rates for incorrect edge inclusion. We demonstrate the proposed approach on various simulated examples as well as on a large gene expression data set from The Cancer Genome Atlas.

## 1 INTRODUCTION

Identifying the complex relationships between molecular entities is central to the understanding of disease biology. The advent of high‐throughput biotechnologies has provided opportunity to study this interplay and considerably stimulated research in this direction. Many studies now exploit high‐throughput molecular data to describe the functional relationships between molecular entities such as genes, proteins, or metabolites.

Graphical models provide a natural basis for the statistical description and analysis of relationships between variables. In applications, interest often lies in the undirected graph that describes the conditional dependence structure among variables. When the joint distribution of the variables is assumed to be Gaussian, this is known to be fully coded in the inverse‐covariance matrix $\Omega =\left\{\omega_{ij}\right\}$ (Dempster, 1972). Precisely, a pair (i,j) of variables with 1≤i<j≤p, will be conditionally independent (given all the remaining variables) when $\omega_{ij}=0$. The present article treats inference of the undirected graph in context of the Gaussian model when the number of variables p is potentially larger than the sample size.

Despite major methodological developments, Bayesian inference for Gaussian graphical models remains challenging. The standard approach casts the problem as a model selection problem, and first requires specification of prior distributions over all possible graphical models and their parameter spaces. Such specification is not straightforward as it is desirable to favor parsimonious models and address the compatibility of priors across models (Carvalho and Scott, 2009; Consonni and La Rocca, 2012). Next, the inference procedure is hindered by the search over a very high‐dimensional model space where the number of possible graphical models grows superexponentially with the number of variables. Full exploration of the model space is, therefore, only possible when the number of variables is very small (say p≤10). In moderate‐dimensional and high‐dimensional settings where p is in the tens, hundreds, or thousands, the model space must generally be searched stochastically (Wang and Li, 2012; Mohammadi and Wit, 2015). However, due to the tremendous size of the model space in such settings, it may be difficult (or impossible) to identify with confidence the graphical model that is best supported by the data. Indeed, many models may almost equally be supported by the data. Accordingly, it is preferable to account for model uncertainty by performing Bayesian model averaging and to infer the graphical structure by selecting edges with the highest marginal posterior probabilities, for example, by exploiting their connection to a Bayesian version of the false discovery rate (Mitra *et al.*, 2013; Baladandayuthapani *et al.*, 2014; Peterson *et al.*, 2015).

To bypass the difficulties associated with the standard approach, this article proposes to use an alternative framework based on directly selecting edges by multiple testing of hypotheses about pairwise conditional independence using closed‐form Bayes factors. These are obtained using the conditional approach of Dickey (1971), in which the prior under the null hypothesis is derived from that of the alternative by conditioning on the null hypothesis. This approach was also adopted by Giudici (1995) to derive a closed‐form Bayes factor for conditional independence. However, the latter relies on elements of the inverse of the sample covariance matrix which is singular when the number of variables is large relative to the sample size. We circumvent this issue and introduce new closed‐form Bayes factors for marginal and conditional independence that are suitable in such settings. Moreover, we show the consistency of the Bayes factors and derive exact tail probabilities from their null distributions to help address the multiplicity problem and control error rates for incorrect edge inclusion. The proposed procedure, available via the R package beam on the CRAN website, is shown to be computationally very efficient, addressing problems with thousands of nodes in just a few seconds.

The next section introduces notations and the Gaussian conjugate (GC) model. Section 3 presents a closed‐form Bayes factor to evaluate the null hypothesis of conditional independence between any two variables and studies its consistency (all results about marginal independence are provided in Appendix S2). Section 4 details graph inference and discusses the multiple testing problem and error control. The performance of the proposed approach is compared to Bayesian and non‐Bayesian methods on simulated data in Section 5. Section 6 illustrates our method on a large gene expression data set from The Cancer Genome Atlas.

## 2 BACKGROUND

### 2.1 Notation

We write $x|\mu ,\Sigma \sim N_{p}\left(\mu ,\Sigma \right)$ to indicate that $x\in R^{p}$ has a multivariate normal distribution with mean μ and positive‐definite covariance matrix $\Sigma ,\Omega |A,\alpha \sim IW_{d}\left(A,\alpha \right)$ to indicate that Ω has an Inverse‐Wishart distribution with scale matrix A and degree of freedom α>d+1, and $\beta \sim \beta \left(b_{1},b_{2}\right)$ to indicate that β has a *β* distribution with shape parameters $b_{1}$ and $b_{2}$. $\Gamma_{d}\left(x\right)$ is the d‐dimensional gamma function, the operator vec denotes the linear transformation that stacks the columns of a matrix into a vector and ⊗ denotes the Kronecker product. We use the subscripts aa,bb,ab, and ba to refer to the submatrices $\Sigma_{aa},\Sigma_{bb},\Sigma_{ab}$, and $\Sigma_{ba}$ of a p×p symmetric matrix Σ whose block‐wise decomposition is implied by a partition of its rows and columns into two disjoint subsets indexed by a⊂{1,…,p} and b={1,…,p}⧹a.

### 2.2 The GC model

Given an n×p observation matrix $Y=\left(Y_{1},\ldots ,Y_{p}\right)$, the GC model is defined by

(1) $$\begin{array}{ccc} & & \mathrm{vec}\left(Y\right)|\Sigma \;\sim \;N_{np}\left(0,\Sigma ⊗I_{n}\right),\\ & & \Sigma |D,\delta \;\sim \;IW_{p}\left(\left(\delta -p-1\right)D,\delta \right), \end{array}$$

with D positive definite, $I_{n}$ the n‐dimensional identity matrix, and δ>p+1. Here, the covariance matrix with Kronecker product structure makes explicit the assumption of independence for the rows of Y and the dependence of its columns via the covariance Σ.

Due to conjugacy, model (1) offers closed‐form Bayesian estimators of the covariance matrix Σ and its inverse $\Omega =\Sigma^{-1}$. The posterior expectation of Σ is

(2) E(Σ∣Y)={(δ−p−1)D+S}∕(δ+n−p−1),

where $S=Y^{T}Y$, and that of its inverse is

(3) $$E\left(\Omega |Y\right)=\left(\delta +n\right){\left\{\left(\delta -p-1\right)D+S\right\}}^{-1}.$$

It is important to note that estimator (2) is a linear shrinkage estimator that is a convex linear combination of the maximum likelihood estimator ${\hat{\Sigma }}_{\text{mle}}=n^{-1}S$ of Σ and E(Σ)=D, with weight α=(δ−p−1)∕(δ+n−p−1)∈(0,1) (Chen, 1979; Hannart and Naveau, 2014). Likewise, estimator (3) is recognized as a ridge‐type estimator of the precision matrix (Kubokawa and Srivastava, 2008; Van Wieringen and Peeters, 2016). The next proposition presents some properties of these two estimators. All proofs are presented in Appendix S4.

Proposition 1 Let estimators (2) and (3) depend on δ with D,n, and p fixed, and denote them by ${\hat{\Sigma }}_{\delta }$ and ${\hat{\Omega }}_{\delta }$, respectively. Then the following properties hold:

(1) $\lim_{\delta \to \infty }{\hat{\Sigma }}_{\delta }=D$;
(2) $\lim_{\delta \to \infty }{\hat{\Omega }}_{\delta }=D^{-1}$;
(3) $\lim_{\delta \to p+1}{\hat{\Sigma }}_{\delta }={\hat{\Sigma }}_{\text{mle}}$;
(4) $\lim_{\delta \to p+1}{\hat{\Omega }}_{\delta }=\left\{\left(n+p+1\right)∕n\right\}{\hat{\Sigma }}_{\text{mle}}^{-1}$, if n>p;
(5) ${\hat{\Sigma }}_{\delta }$ and ${\hat{\Omega }}_{\delta }$ are positive definite.

Additionally, the asymptotic properties of estimators (2) and (3) when n→∞ are the same as those of the maximum likelihood estimators ${\hat{\Sigma }}_{\text{mle}}$ and ${\hat{\Sigma }}_{\text{mle}}^{-1}$ of Σ and Ω. Proposition 2 summarizes.

Proposition 2 Let estimator (2) and (3) depend on n with D,δ, and p be fixed, and denote them by ${\hat{\Sigma }}_{n}$ and ${\hat{\Omega }}_{n}$, respectively. Then the following properties hold:

(1) $\lim_{n\to \infty }{\hat{\Sigma }}_{n}={\hat{\Sigma }}_{\text{mle}}$;
(2) $\lim_{n\to \infty }{\hat{\Omega }}_{n}={\hat{\Sigma }}_{\text{mle}}^{-1}$.

### 2.3 Choice of hyperparameters

In model (1), the prior matrix D represents the prior expectation of Σ. It may also be interpreted as the shrinkage target toward which the maximum likelihood estimator of the covariance matrix is shrunk, since the posterior expectation of Σ is a linear shrinkage estimator. For these reasons, D can be chosen to encourage estimator (2) to have specific structures (eg, autoregressives or low ranks). Ideally, in such cases the matrix D should be parameterized by a low‐dimensional vector of hyperparameters that are interpretable and for which prior knowledge exists. As often this knowledge is absent, it is common to choose $D=I_{p}$. Throughout this paper, we use $D=I_{p}$ and standardize the n×p observation matrix Y so that for $1\leq j\leq p,Y_{j}^{T}1_{n}=0$ and $Y_{j}^{T}Y_{j}∕n=1$, where $1_{n}$ is an n×1 vector whose elements are all equal to 1.

The other hyperparameter δ clearly acts as a regularization parameter (see Equations (2) and (3)) and its value must therefore be chosen carefully. Following Chen (1979) and Hannart and Naveau (2014), we use empirical Bayes and estimate δ by the value $\hat{\delta }$ maximizing the marginal (or integrated) likelihood of the model (see Appendix S2). We are referring the reader to Hannart and Naveau (2014, Section 2.3) for the proof that the asymptotic properties of estimator (2) and (3) (Proposition 1) hold when $\delta =\hat{\delta }$.

## 3 BAYES FACTORS

### 3.1 Bayes factor for conditional independence

In this section we derive an analytic expression for the Bayes factor evaluating the null hypothesis of conditional independence between two variables in context of model (1). For ease of notation, we define F=(δ−p−1)D and T=F+S. We wish to evaluate the null hypothesis of conditional independence, denoted $H_{0,ij}$, between two coordinates i and j,1≤i<j≤p. We test $H_{0,ij}:\omega_{ij}=0$ against the alternative hypothesis $H_{1,ij}:\omega_{ij}\neq 0$, where $\omega_{ij}$ is the (*i*,*j*)th element of Ω. The Bayes factor evaluating evidence in favor of $H_{1,ij}$ is

(4) $${\mathrm{BF}}_{ij}=\frac{\int p_{1}\left(Y|\Sigma \right)p_{1}\left(\Sigma \right)d\Sigma }{\int p_{0}\left(Y|\Sigma^{0}\right)p_{0}\left(\Sigma^{0}\right)d\Sigma^{0}},$$

where, by definition, $\Sigma_{0}$ is such that $\omega_{ij}=0$.

Giudici (1995) showed that (4) could be obtained in closed form by reparameterizing the GC model and defining a compatible prior under the null hypothesis using the approach of Dickey (1971). However, the proposed Bayes factor does not exist in high‐dimensional settings where p>n because it depends on elements of $S^{-1}$. This problem is here circumvented by factorizing the joint likelihood of the observed data as $p\left(Y|\Sigma \right)=p\left(Y_{b}|\Sigma_{bb}\right)p\left(Y_{a}|Y_{b},B_{a|b},\Sigma_{aa.b}\right)$, the product of a marginal and conditional likelihood. This factorization arises from the partition of $Y=\left[Y_{a},Y_{b}\right]$ into two disjoint subsets indexed by a={i,j} and b=V⧹a. The quantity $B_{a|b}=\Sigma_{bb}^{-1}\Sigma_{ba}$ represents the matrix of regression coefficients obtained when regressing the variables indexed by a onto the variables indexed by b, whereas $\Sigma_{aa.b}=\Sigma_{aa}-\Sigma_{ab}\Sigma_{bb}^{-1}\Sigma_{ba}$ denotes the residual covariance matrix.

The factorization of the likelihood allows conveniently to simplify (4). Using the change of variable from $\left(\Sigma_{aa},\Sigma_{ab},\Sigma_{bb}\right)$ to $\left(\Sigma_{aa.b},B_{a|b},\Sigma_{bb}\right)$ together with the fact that $\Sigma_{bb}$ is independent of $\left(B_{a|b},\Sigma_{aa.b}\right)$, most nuisance parameters are integrated out and (4) becomes

(5) $${\mathrm{BF}}_{ij}=\frac{\iint p_{1}\left(Y_{a}|Y_{b},B_{a|b},\Sigma_{aa.b}\right)p_{1}\left(B_{a|b},\Sigma_{aa.b}\right)dB_{a|b}d\Sigma_{aa.b}}{\iint p_{0}\left(Y_{a}|Y_{b},B_{a|b},\Sigma_{aa.b}^{0}\right)p_{0}\left(B_{a|b},\Sigma_{aa.b}^{0}\right)dB_{a|b}d\Sigma_{aa.b}^{0}}.$$

Note that by the standard properties of the multivariate normal and inverse‐Wishart distributions (Gupta and Nagar, 2000, Theorems 2.3.12 and 3.3.9) the densities under the alternative model are

(6) $$\begin{array}{ccc} \mathrm{vec}\left(Y_{a}\right)|Y_{b},B_{a|b},\Sigma_{aa.b} & \sim & N_{n\times 2}\left(\mathrm{vec}\left(Y_{b}B_{a|b}\right),\Sigma_{aa.b}⊗I_{n}\right),\\ \mathrm{vec}\left(B_{a|b}\right)|\Sigma_{aa.b} & \sim & N_{\left(p-2\right)\times 2}\left(\mathrm{vec}\left(F_{a|b}\right),\Sigma_{aa.b}⊗F_{bb}^{-1}\right),\\ \Sigma_{aa.b} & \sim & IW_{2}\left(F_{aa.b},\delta \right), \end{array}$$

where $F_{a|b}=F_{bb}^{-1}F_{ba}$ and $F_{aa.b}=F_{aa}-F_{ab}F_{bb}^{-1}F_{ba}$. Therefore, the simplification of Bayes factor (4) intuitively tells us that evaluating the conditional independence between any two coordinates within the p‐dimensional GC model (1) is equivalent to evaluating the diagonality of the residual covariance matrix in a bivariate regression model.

To obtain (5) in closed form we, similar to Giudici (1995), define a compatible prior for $\left(B_{a|b},\Sigma_{aa.b}\right)$ under the null hypothesis $H_{0,ij}$ using the conditional approach of Dickey (1971). Precisely, the prior density under $H_{0,ij}$ is derived from that under $H_{1,ij}$ by conditioning on $H_{0,ij}$. The densities under the null model are therefore

(7) $$\begin{array}{ccc} \mathrm{vec}\left(Y_{a}\right)|Y_{b},B_{a|b},\Sigma_{aa.b}^{0} & \sim & N_{n\times 2}\left(\mathrm{vec}\left(Y_{b}B_{a|b}\right),\Sigma_{aa.b}^{0}⊗I_{n}\right),\\ \mathrm{vec}\left(B_{a|b}\right)|\Sigma_{aa.b}^{0} & \sim & N_{\left(p-2\right)\times 2}\left(\mathrm{vec}\left(F_{a|b}\right),\Sigma_{aa.b}^{0}⊗F_{bb}^{-1}\right),\\ p_{0}\left(B_{a|b},\Sigma_{aa.b}^{0}\right) & = & p_{1}\left(B_{a|b},\Sigma_{aa.b}|H_{0,ij}\right)\\ & = & \frac{p_{1}\left(B_{a|b},\Sigma_{aa.b},H_{0,ij}\right)}{\iint p_{1}\left(B_{a|b},\Sigma_{aa.b},H_{0,ij}\right)dB_{a|b}d\Sigma_{aa.b}}, \end{array}$$

where $\Sigma_{aa.b}^{0}$ is such that $\omega_{ij}=0$.

We now state the main result of this section.

Lemma 1 Assume (5) holds with densities defined by (6) and (7). Then the Bayes factor in favor of $H_{1,ij}$ is

$${\mathrm{BF}}_{ij}=k_{1}\left(\delta ,n\right)\frac{{\left(1-r_{g_{ij}}^{2}\right)}^{\delta /2}}{{\left(1-r_{q_{ij}}^{2}\right)}^{\left((\delta +n)/2\right)}}{\left(\frac{g_{ii}g_{jj}}{q_{ii}q_{jj}}\right)}^{1/2},$$

with

$$k_{1}\left(\delta ,n\right)=\frac{\Gamma \left((\delta +n)/2\right)\Gamma \left((\delta +n-1)/2\right)\Gamma^{2}\left((\delta +1)/2\right)}{\Gamma \left(\delta /2\right)\Gamma \left((\delta -1)/2\right)\Gamma^{2}\left((\delta +n+1)/2\right)},$$

$$r_{g_{ij}}=\frac{g_{ij}}{\sqrt{g_{ii}g_{jj}}},r_{q_{ij}}=\frac{q_{ij}}{\sqrt{q_{ii}q_{jj}}},F_{aa.b}=\left[\begin{array}{cc} g_{ii} & g_{ij}\\ g_{ij} & g_{jj} \end{array}\right],T_{aa.b}=\left[\begin{array}{cc} q_{ii} & q_{ij}\\ q_{ij} & q_{jj} \end{array}\right].$$

Remark 1 In Lemma 1, the quantities $g_{ii}$ and $q_{ii}$ (resp., $g_{jj}$ and $q_{jj}$) can be thought of representing prior and posterior partial variances for coordinate i (resp., j), whereas $r_{g_{ij}}$ and $r_{q_{ij}}$ can be thought of representing prior and posterior partial correlations.

Remark 2 The Bayes factor proposed by Giudici (1995, Lemma 3), in contrast to Lemma 1, defines the quantities $g_{ij}$ and $q_{ij}$ such that the matrices $F_{aa.b}=\left\{g_{ij}\right\}$ and $F_{aa.b}+S_{aa.b}=\left\{q_{ij}\right\}$, with $S_{aa.b}=S_{aa}-S_{ab}S_{bb}^{-1}S_{ba}$. Note that here $S_{aa.b}$ only exists when $S_{bb}$ is invertible (ie, when n is large relatively to p) whereas $T_{aa.b}=T_{aa}-T_{ab}T_{bb}^{-1}T_{ba}$ defined in Lemma 1 exists even when p>n because T is always positive definite (a consequence of Proposition 1).

Remark 3 Standard matrix algebra (Gupta and Nagar, 2000, Theorem 1.2.3.(v)) tells us that $F_{aa.b}={\left\{{\left(F^{-1}\right)}_{aa}\right\}}^{-1}$ and $T_{aa.b}={\left\{{\left(T^{-1}\right)}_{aa}\right\}}^{-1}$. This means that the elements of the 2×2 matrices $F_{aa.b}$ and $T_{aa.b}$ can, respectively, be obtained from the elements of $F^{-1}$ and $T^{-1}$. The computation of the Bayes factor in Lemma 1 for all pairs of variables (i,j) hence boils down to computing $F^{-1}$ and $T^{-1}$.

### 3.2 Consistency

In this section, we consider the selection consistency of the Bayes factor defined in Lemma 1. A Bayes factor is said to be consistent when $\lim_{n\to \infty }{\mathrm{BF}}_{ij}=0$ if $H_{0,ij}$ is true and $\lim_{n\to \infty }{\mathrm{BF}}_{ij}=\infty$ if $H_{1,ij}$ is true (Wang and Maruyama, 2016). In other words, the consistency property means that the true hypothesis will be selected when enough data are provided. We now state the following result.

Lemma 2 If the sample correlation matrix has a limit as n→∞ that is positive definite, then the Bayes factor ${\mathrm{BF}}_{ij}$ is consistent in selection.

## 4 GRAPH STRUCTURE RECOVERY

### 4.1 Inference by multiple testing

We propose to infer the conditional independence graph by multiple testing of hypotheses using the Bayes factor introduced in the previous section. Precisely, we propose to infer the edge set $E_{U}=\left\{\left(i,j\right)|\omega_{ij}\neq 0\right\}$ of the undirected graph $U=\left(V,E_{U}\right)$ with vertex set V by evaluating $H_{0,ij}:\omega_{ij}=0$ (absence of an edge) vs. $H_{1,ij}:\omega_{ij}\neq 0$ (presence of an edge) separately for each pair (i,j) of variables.

On the whole, the multiple testing approach consists in translating the pattern of rejected hypotheses into a graph. The approach is justified by the fact that, for the undirected graph, the conditioning sets in the pairwise independence statements do not depend on the structure of the graph (Drton and Perlman, 2007). This means that these statements can be evaluated individually by hypothesis testing. Here, these tests are carried out separately using model (1) that encodes the complete undirected graph where no independence structure is imposed.

### 4.2 Scaled Bayes factors

To infer the graph structure it is necessary to compare Bayes factors between all p(p−1)∕2 pairs of variables. However, the Bayes factor defined in Lemma 1 is not scale‐invariant (due to its last term) and, hence, not comparable between different pairs of variables. In light of this, we define a scaled version of this Bayes factor that can more appropriately rank edges of graph U. Corollary 1 summarizes.

Corollary 1 The scaled Bayes factor in favor of $H_{1,ij}$ is

$${\mathrm{sBF}}_{ij}=k_{1}\left(\delta ,n\right)\frac{{\left(1-r_{g_{ij}}^{2}\right)}^{\delta /2}}{{\left(1-r_{q_{ij}}^{2}\right)}^{\left((\delta +n)/2\right)}},$$

with quantities defined as in Lemma 1.

Remark 4 When the prior matrix $D=I_{p}$ (absence of prior knowledge), then $r_{g_{ij}}=0$ and the ordering provided by the scaled Bayes factor in Corollary 1 for all pairs (i,j) is identical to the ordering provided by the square of the posterior partial correlation $r_{q_{ij}}$. This means that the graph selected when using a thresholding rule on the Bayes factors is the same as that obtained using the equivalent thresholding rule on the posterior correlations.

### 4.3 Multiplicity adjustment and error control

To address the multiplicity problem, we propose to use the tail or error probability associated with the null distribution of each scaled Bayes factor. The tail probability is closely related to the notion of a *P*‐value: the Bayes factor is treated as a random variable and its distribution, which follows that of the random data, is used to make a probability statement about its observed value. Then, to recover the structure of a graph, the tail probabilities obtained from all p(p−1)∕2 comparisons are adjusted using standard multiplicity correction procedures to control, say, the family‐wise error or false discovery rates (Goeman and Solari, 2014).

In the following, we study the conditional null distribution of the Bayes factor defined in Corollary 1. The conditional null distribution here refers to the distribution that would be obtained by shuffling or permuting labels of the observations (Jiang *et al.*, 2017). Under this scheme, we shall define $Pr\left({\mathrm{sBF}}_{ij}>b\right)$ the probability of observing a value for the scaled Bayes factor that is larger than b. Next, we show that this tail probability can be obtained analytically without the need of a permutation algorithm, thus providing a computational advantage. Before, we state three results which will be used in our argumentation.

Proposition 3 Suppose $\Phi \sim W_{2}\left(\Sigma ,d\right)$, where

$$\Phi =\left(\begin{array}{cc} \phi_{1}^{2} & \phi_{1}\phi_{2}\varphi \\ \phi_{1}\phi_{2}\varphi & \phi_{2}^{2} \end{array}\right)\;\text{and}\;\Sigma =\left(\begin{array}{cc} \sigma_{1}^{2} & \sigma_{1}\sigma_{2}\rho \\ \sigma_{1}\sigma_{2}\rho & \sigma_{2}^{2} \end{array}\right)$$

are parametrized in terms of their correlations −1≤φ≤1 and −1≤ρ≤1. Then,

$$\left(\varphi^{2}|\rho =0\right)\sim \beta \left(1∕2,\left(d-1\right)∕2\right).$$

Proposition 4 The following equality holds:

$$\begin{array}{ccc} & & Y_{a}^{T}Y_{a}-{\bar{B}}_{a|b}^{T}\left(Y_{b}^{T}Y_{b}+F_{bb}\right){\bar{B}}_{a|b}+F_{ab}F_{bb}^{-1}F_{ba}\\ & & ={\left(Y_{a}-Y_{b}F_{a|b}\right)}^{T}{\left(I_{n}+Y_{b}F_{bb}^{-1}Y_{b}^{T}\right)}^{-1}\left(Y_{a}-Y_{b}F_{a|b}\right), \end{array}$$

where ${\bar{B}}_{a|b}={\left(Y_{b}^{T}Y_{b}+F_{bb}\right)}^{-1}\left(Y_{b}^{T}Y_{a}+F_{ba}\right)$.

Proposition 5 Let $\Sigma_{aa.b}$ be fixed. Then, according to model (6), we have

$${\left(Y_{a}-Y_{b}F_{a|b}\right)}^{T}{\left(I_{n}+Y_{b}F_{bb}^{-1}Y_{b}^{T}\right)}^{-1}\left(Y_{a}-Y_{b}F_{a|b}\right)\sim W_{2}\left(\Sigma_{aa.b},n\right).$$

The only term of the Bayes factor that depends on the data is $r_{q_{ij}}=q_{ij}{\left(q_{ii}q_{jj}\right)}^{-1∕2}$, where, we recall, $q_{ij}$ is such that $T_{aa.b}=\left\{q_{ij}\right\}$. Proposition 4 suggests that $T_{aa.b}=F_{aa.b}+Z$, with $Z={\left(Y_{a}-Y_{b}F_{a|b}\right)}^{T}{\left(I_{n}+Y_{b}F_{bb}^{-1}Y_{b}^{T}\right)}^{-1}\left(Y_{a}-Y_{b}F_{a|b}\right)$. Hence,

$$r_{q_{ij}}=\frac{{\left(g_{ii}g_{jj}\right)}^{1∕2}r_{g_{ij}}+{\left(z_{ii}z_{jj}\right)}^{1∕2}r_{z_{ij}}}{{\left(g_{ii}+z_{ii}\right)}^{1∕2}{\left(g_{jj}+z_{jj}\right)}^{1∕2}},$$

where $Z=\left\{z_{ij}\right\}$ and $r_{z_{ij}}=z_{ij}{\left(z_{ii}z_{jj}\right)}^{-1∕2}$. This means that $Pr\left\{{\mathrm{sBF}}_{ij}>b\right\}=Pr\left\{r_{z_{ij}}^{2}>c\right\}$, where c is a quantity that depends on $\left\{\delta ,n,g_{ii},g_{jj},r_{g_{ij}},z_{ii},z_{jj}\right\}$. Propositions 3 and 5 imply that $Z\sim W_{2}\left(\Sigma_{aa.b},n\right)$ and $r_{z_{ij}}^{2}|H_{0,ij}\sim \beta \left(1∕2,\left(n-1\right)∕2\right)$. Therefore, the tail probability of the Bayes factor can be computed exactly using β(1∕2,(n−1)∕2). We remark that the definition of the type 1 error is conditioning on $\left\{\delta ,n,g_{ii},g_{jj},r_{g_{ij}},z_{ii},z_{jj}\right\}$.

## 5 NUMERICAL EXPERIMENTS

### 5.1 Comparison to Bayesian methods

In this section, we compare the performance of our approach with other Bayesian methods. For computational reasons, we consider a moderate‐dimensional problem. We generate 50 datasets of size n∈{25,50,100} from a multivariate Gaussian distribution with mean vector 0 and 50×50 inverse‐covariance matrix Φ. The matrix Φ is a sparse matrix which we generate from a G‐Wishart distribution with scale matrix equal to the identity and b=4 degrees of freedom (using the function bdgraph.sim of R package BDgraph). Four different graph structures are considered, namely the *band*, *cluster*, *hub*, and *random* structures, which we illustrate in Figure S1.

We compare our method to two sampling‐based approaches based on the birth‐death and reversible jump Markov chain Monte Carlo (MCMC) algorithms, developed by Mohammadi and Wit (2015; 2017), using 100 000 sweeps and a burn‐in period of 50 000 updates. We also consider the method of Schwaller *et al.* (2017) that offers closed‐form inference within the class of tree‐structured graphical models. For each method we obtain the marginal posterior probabilities of edge inclusion, either via the sampling algorithm or exactly.

To evaluate performance we report the area under the receiver operating characteristic (ROC) curve, which depicts the true positive rate *TP*/(*TP *+ *FN*) as a function of the false positive rate *FP*/(*FP*  + *TP*), overall possible thresholds on the marginal posterior probabilities of edge inclusion (or tail probabilities in case of our method). Here, TP,FP, and FN denote the number of true positives, false positives, and false negatives, respectively. We also report the area under the precision recall (PR) curve, which depict the precision *TP*/(*TP* + *FP*) as a function of the true positive rate (also named recall).

Table 1 summarizes simulation results. It shows that our method performs well compared to other Bayesian methods in recovering the different graph structures. For instance, our method often achieves the largest areas under the ROC and PR curves for different graph structures and sample sizes. Moreover, a marked improvement is observed in cases where the sample size is small (n=25) with respect to p. The results also show nonnegligible differences in performance between the birth‐death and reversible jump MCMC algorithms, which suggests that performance can be affected by the choice of sampling algorithm.

**Table 1:** Average and SD (in parenthesis) of areas under the ROC and PR curves over the simulated datasets, as a function of the true graph structure and sample size n

		Band structure		Cluster structure	
*n*	Methods	${\mathrm{AUC}}_{\mathrm{ROC}}$	${\mathrm{AUC}}_{\mathrm{PR}}$	${\mathrm{AUC}}_{\mathrm{ROC}}$	${\mathrm{AUC}}_{\mathrm{PR}}$
100	beam	**0.89 (0.02)**	0.65 (0.03)	**0.80 (0.02)**	**0.54 (0.03)**
100	bdmcmc	**0.89 (0.03)**	**0.67 (0.03)**	0.79 (0.02)	0.51 (0.04)
100	rjmcmc	0.88 (0.03)	0.63 (0.05)	0.78 (0.03)	0.50 (0.04)
100	saturnin	**0.89 (0.02)**	0.61 (0.04)	0.77 (0.02)	0.53 (0.04)
50	beam	**0.84 (0.03)**	**0.53 (0.04)**	**0.73 (0.02)**	**0.39 (0.04)**
50	bdmcmc	0.82 (0.03)	0.51 (0.06)	0.72 (0.03)	0.37 (0.04)
50	rjmcmc	0.81 (0.03)	0.47 (0.05)	0.72 (0.02)	0.35 (0.04)
50	saturnin	0.82 (0.02)	0.44 (0.04)	0.68 (0.02)	0.33 (0.04)
25	beam	**0.78 (0.04)**	**0.39 (0.05)**	**0.66 (0.03)**	**0.24 (0.04)**
25	bdmcmc	0.75 (0.04)	0.32 (0.05)	0.65 (0.03)	0.23 (0.03)
25	rjmcmc	0.75 (0.04)	0.27 (0.05)	0.64 (0.03)	0.22 (0.03)
25	saturnin	0.73 (0.03)	0.28 (0.05)	0.58 (0.02)	0.15 (0.02)

		Hub structure		Random structure	
100	beam	0.88 (0.03)	0.62 (0.03)	**0.87 (0.03)**	0.65 (0.03)
100	bdmcmc	0.89 (0.02)	**0.67 (0.04)**	0.86 (0.03)	**0.66 (0.03)**
100	rjmcmc	0.89 (0.02)	0.65 (0.05)	0.85 (0.03)	0.65 (0.04)
100	saturnin	**0.92 (0.01)**	0.63 (0.02)	0.86 (0.02)	0.59 (0.02)
50	beam	0.84 (0.03)	**0.53 (0.03)**	**0.83 (0.03)**	**0.56 (0.04)**
50	bdmcmc	0.84 (0.03)	0.52 (0.05)	0.81 (0.03)	0.53 (0.05)
50	rjmcmc	0.84 (0.03)	0.48 (0.06)	0.80 (0.03)	0.49 (0.06)
50	saturnin	**0.86 (0.02)**	0.48 (0.03)	**0.83 (0.02)**	0.47 (0.03)
25	beam	**0.80 (0.03)**	**0.42 (0.04)**	**0.79 (0.03)**	**0.43 (0.05)**
25	bdmcmc	0.79 (0.04)	0.32 (0.05)	0.75 (0.02)	0.33 (0.05)
25	rjmcmc	0.77 (0.04)	0.27 (0.04)	0.74 (0.03)	0.30 (0.05)
25	saturnin	**0.80 (0.03)**	0.35 (0.04)	0.77 (0.02)	0.35 (0.04)

Abbreviation: AUC, area under curve; PR, precision recall; ROC, receiver operating characteristic.

beam, our method; bdmcmc and rjmcmc, methods of Mohammadi and Wit (2015); saturnin, method of Schwaller et al. (2017); ${\mathrm{AUC}}_{\mathrm{ROC}}$, area under the ROC curve; ${\mathrm{AUC}}_{\mathrm{PR}}$ area under the PR curve. Best performances are boldfaced.

Overall, the simulation results demonstrate that our method can recover various graphical structures at least as accurately as other Bayesian approaches at a very low computation cost (see Figure S2). Our method achieves generally a greater area under the PR curve than others. The present results also confirm that obtained by Schwaller *et al.* (2017), namely, the relative good performance of tree‐structured graphical models compared to sampling‐based approaches despite stronger restrictions on the class of graphical models. However, the performance of the approach can degrade in some cases (eg, cluster structures).

### 5.2 Comparison to non‐Bayesian methods

The performance of the proposed method is compared in higher dimensional settings to non‐Bayesian approaches that carry out graphical model selection via multiple testing. We generate 50 datasets of size n=100 from a p‐dimensional Gaussian distribution mean vector 0 and inverse‐covariance matrix Ψ. Throughout the simulation, we fix the sample size n=100 and vary of the dimensionality p∈{200,500,1000}. We consider four different sparse precision matrices corresponding to different graph structures (similar to those illustrated in Figure S1): (a) $\Psi_{p}^{\text{band}}$ is a tridiagonal matrix; (b) $\Psi_{p}^{\text{cluster}}$ is a block diagonal matrix whose blocks are sparse matrices of size 20 where off‐diagonal entries are nonzero with probability 0.1; (c) $\Psi_{p}^{\text{hub}}$ is a block diagonal matrix whose blocks are sparse matrices of size 20 where only the off‐diagonal entries in the first row and column are nonzero; and (d) $\Psi_{p}^{\text{random}}$ is obtained by randomly permuting the rows and columns of $\Psi_{p}^{\text{band}}$. For all matrices nonzero entries are generated independently from a uniform distribution on [−1,1] and positive definiteness is ensured by adding a constant to the diagonal so the minimum eigenvalue is equal to 0.1.

We compare our method to that of Schäfer and Strimmer (2005) that is based on a linear shrinkage estimator of the covariance matrix (Ledoit and Wolf, 2004) and a mixture model for false discovery rate estimation (Strimmer, 2008). We also consider the asymptotic normal thresholding method of Ren *et al.* (2015). For both methods we obtain *P* values associated with the estimated partial correlations, whereas for our method we use the tail probabilities associated with the Bayes factor defined in Corollary 1 for all pairs of variables.

As in Section 5.1, we evaluate performance using the areas under the ROC and PR curves.

Table 2 shows that the proposed method performs well in recovering large graphical structures compared to non‐Bayesian methods. It achieves comparable areas under the ROC and PR curves as other methods for different problem sizes. However, in the case of hub structures the proposed method performs better.

**Table 2:** Average and SD (in parenthesis) areas under the ROC and PR curves over the simulated datasets, and as a function of the true graph structure and sample size n

		Band structure		Cluster structure	
*p*	Methods	${\mathrm{AUC}}_{\mathrm{ROC}}$	${\mathrm{AUC}}_{\mathrm{PR}}$	${\mathrm{AUC}}_{\mathrm{ROC}}$	${\mathrm{AUC}}_{\mathrm{PR}}$
200	beam	0.88 (0.01)	0.55 (0.02)	**0.91 (0.01)**	0.58 (0.01)
200	genenet	**0.89 (0.01)**	**0.57 (0.02)**	**0.91 (0.01)**	0.59 (0.01)
200	fastggm	0.87 (0.01)	**0.57 (0.02)**	0.89 (0.01)	**0.60 (0.02)**
500	beam	**0.91 (0.01)**	0.58 (0.01)	**0.89 (0.01)**	0.50 (0.01)
500	genenet	**0.91 (0.01)**	0.60 (0.01)	**0.89 (0.01)**	**0.52 (0.01)**
500	fastggm	0.90 (0.01)	**0.61 (0.01)**	0.85 (0.01)	0.49 (0.01)
1000	beam	**0.88 (0.01)**	0.49 (0.01)	**0.90 (0.00)**	0.48 (0.01)
1000	genenet	**0.88 (0.01)**	0.49 (0.01)	**0.90 (0.00)**	**0.49 (0.01)**
1000	fastggm	0.87 (0.01)	**0.51 (0.01)**	0.87 (0.00)	0.48 (0.01)

		Hub structure		Random structure	
200	beam	**0.90 (0.01)**	**0.56 (0.01)**	**0.86 (0.01)**	0.43 (0.02)
200	genenet	0.85 (0.01)	0.21 (0.03)	**0.86 (0.01)**	**0.47 (0.02)**
200	fastggm	0.87 (0.01)	0.46 (0.02)	0.85 (0.01)	**0.47 (0.02)**
500	beam	**0.92 (0.01)**	**0.54 (0.01)**	**0.82 (0.01)**	**0.35 (0.01)**
500	genenet	0.90 (0.00)	0.43 (0.01)	**0.82 (0.01)**	0.34 (0.01)
500	fastggm	0.88 (0.01)	0.44 (0.01)	0.81 (0.00)	0.34 (0.01)
1000	beam	**0.93 (0.00)**	**0.54 (0.01)**	**0.77 (0.00)**	**0.22 (0.01)**
1000	genenet	0.92 (0.00)	0.49 (0.01)	**0.77 (0.00)**	0.21 (0.01)
1000	fastggm	0.89 (0.00)	0.44 (0.01)	**0.77 (0.00)**	**0.22 (0.01)**

Abbreviation: AUC, area under curve; PR, precision recall; ROC, receiver operating characteristic.

beam, our method; saturnin, method of Schwaller et al. (2017); genenet, method of Schäfer and Strimmer (2005); fastggm, method of Ren et al. (2015); ${\mathrm{AUC}}_{\mathrm{ROC}}$, area under the ROC curve; ${\mathrm{AUC}}_{\mathrm{PR}}$ area under the PR curve. Best performances are boldfaced.

**Figure 1:**
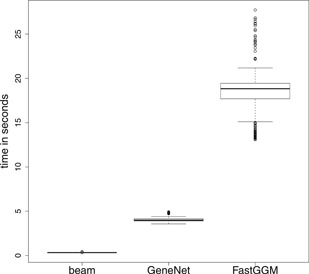
Running time in seconds (assessed on 3.40 GHz Intel Core i7‐3770 CPU) for each method when p=1000

Besides recovering accurately the different graphical structures, Figure 1 shows that the proposed method is the fastest. When p=1000, the average computational time is less than a second whereas contenders are 5 to 20 times slower.

### 5.3 Robustness

We here carry out simulations to assess the robustness of the proposed method to model misspecification as compared to the Bayesian and non‐Bayesian contenders of Sections 5.1 and 5.2. We explore three scenarios where the data are (a) multivariate‐*t* distributed, (b) Gaussian contaminated, and (c) log‐Gaussian distributed. Scenarios 1 and 2 are as in Lin *et al.* (2016), whereas scenario 3 introduces more skewness. For each scenario, we fix p=50 and generate 50 datasets of size n∈{25,50,100} using the same four graphical structures (and inverse‐covariance matrices) considered in Section 5.1.

Results are provided in Appendices S6 to S8. ROC and PR curves show that the proposed method is fairly robust to model misspecification. All methods under consideration logically suffer from model misspecification, however, the proposed method keeps an edge over contenders. Results also suggest that the performance of sampling‐based Bayesian methods, which explore the model space, is most affected by model misspecification.

## 6 GENE NETWORK IN GLIOBLASTOMA MULTIFORME

We illustrate our method on a large gene expression data set on glioblastoma multiforme from The Cancer Genome Atlas. Glioblastoma multiforme is an aggressive form of brain tumor in adults associated with poor prognosis. The data comprise measurements (level 3 normalized; Agilent 244K platform) of 14 827 genes on 532 patients. A small subset of the data were analyzed in Leday *et al.* (2017). Instead, we here characterize globally the conditional independence structure between all 14 827 genes.

**Figure 2:**
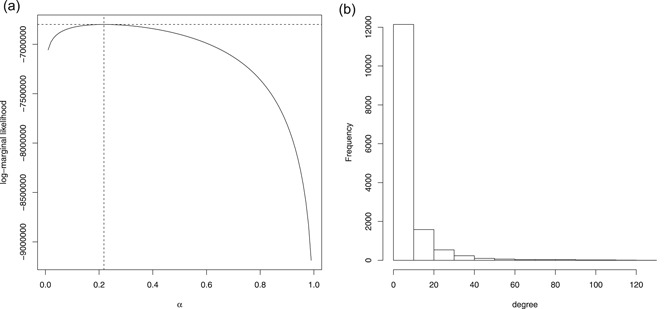
A, Log‐marginal likelihood of the GC model as a function of α=(δ−p−1)∕(δ+n−p−1). The vertical and horizontal dotted lines indicates the location of the optimum. B, Degree distribution of the conditional independence graph. GC, Gaussian conjugate

Figure 2A displays the log‐marginal likelihood of model (1) as a function of the prior parameter α when $D=I_{p}$. Using the empirical Bayes estimate of α we computed the Bayes factors and their associated tail probabilities for all pair of variables. These computations took 90 seconds overall on 3.40 GHz Intel Core i7‐3770 CPU without parallel schemes, which is remarkable for a graph with a total number of 109 912 551 possible edges.

The conditional independence graph identified by controlling the family‐wise error rate at 10% using the conservative Bonferroni procedure consists of 46,071 edges (0.042% of the total number of edges). Edge degree varies from 0 to 127 with 9675 genes having nonzero degrees. The degree distribution seems to follow an exponential distribution (see Figure 2A), thereby indicating that a relative small number of genes have a large number of links.

**Figure 3:**
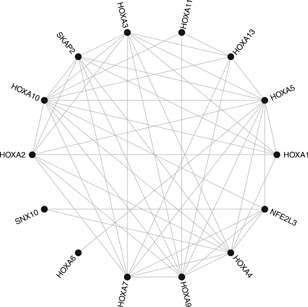
Example of a densely connected gene subgraph identified by the clustering algorithm of Blondel et al. (2008)

Because it is difficult to visualize the graph in its entirety, we identify groups of densely connected nodes using the algorithm of Blondel *et al.* (2008) implemented in the R package igraph (Csardi and Nepusz, 2006). The algorithm identifies a partition that yields an overall modularity score equal to 0.91. The modularity score measures the quality of a division of a graph into subgraphs. Its maximal value being 1, the identified partition presents a high modularity and suggests the presence of densely interconnected groups of nodes in the conditional independence graph. To illustrate this, we report a subgraph in Figure 3 that has been identified by the clustering algorithm and corresponds to the HOXA gene family. The HOX gene family is known to be involved in the development of human cancers (Bhatlekar *et al.*, 2014), including glioblastoma. The HOXA13 gene has for instance been advanced as potential diagnostic marker for glioblastoma (Duan *et al.*, 2015) and the role of HOXA9 gene in cell proliferation, apoptosis, and drug resistance are under active research (Costa *et al.*, 2010; Gonçalves *et al.*, 2016).

## 7 DISCUSSION

This article introduced a Bayesian method to infer the conditional (and marginal) independence structure between variables by multiple testing, which bypasses the exploration of the model space and can easily tackle very large problems with thousands of variables. In extensive simulations, the proposed method was shown to perform at least as good as Bayesian and non‐Bayesian contenders while being orders of magnitude faster. The method was illustrated on a large gene expression data set comprising 14 827 genes.

The proposed method has the advantage of being extremely fast and providing explicit control of the type I error. Moreover, it facilitates the incorporation of (different types of) prior information, which is more difficult in a non‐Bayesian setting. For example, the proposed method can incorporate prior marginal and partial correlations via the hyperparameter D, prior probabilities or odds ratios via the Bayes factors, as well as prior group information (eg, pathways) via the multiple testing procedure (Ramdas *et al.*, 2018).

The main limitation of the proposed method relates to estimation. The proposed approach is based on a simple linear shrinkage estimator that does not perform as well as sparse estimators in sparse settings, unless prior knowledge is used (see Appendix S9). Moreover, the multiple testing procedure identifies the most important edges but does not necessarily yield a graphical model that fits well the data (Drton and Perlman, 2007) because the emphasis is on type I error control rather than goodness‐of‐fit.

We foresee several promising extensions of the proposed approach. The Bayes factors proposed in this paper can be used for differential network analysis in which the goal is to identify edges that are in common or specific to predefined groups of samples. Provided that samples between groups are independent, the Bayes factors can simply be multiplied across groups so as to obtain new Bayes factors that provide evidence toward the presence or absence of a common edge. Being symmetric, the Bayes factors can also be inverted before being multiplied so as to evaluate more complex hypotheses, for example, edge losses or gains in a two‐group comparison. Last, it would be interesting to derive the Bayes factor in a regression framework so as to compare them with that of Zhou and Guan (2018).

## ACKNOWLEDGMENTS

This research was supported by the Medical Research Council grant number MR/M004421 and core funding number MRC_MC_UP_0801/1. The authors wish to thank Ilaria Speranza for helpful comments on the manuscript and improving largely the software. The authors also wish to thank Catalina Vallejos and Leonardo Bottolo for helpful discussions.

## Supporting information

Supplementary Information

Supplementary Information
