# Supplementary material for: Fast Bayesian inference in large Gaussian graphical models
Source: Biometrics. 2019 May 6;75(4):1288–98. doi: 10.1111/biom.13064 (PMC6916355; doi:10.1111/biom.13064)
Supplement: Supplementary file 1 — Supplementary Information [file BIOM-75-1288-s001.pdf]

Supporting Information for

Fast Bayesian inference in large Gaussian graphical models

by

Gwenaël G.R. Leday and Sylvia Richardson

MRC Biostatistics Unit, University of Cambridge, United Kingdom

Contents

Web Appendix A: Empirical Bayes **2**

Web Appendix B: Bayes factor for marginal independence **2**

Web Appendix C: Tail probabilities **6**

Web Appendix D: Proofs **6**

Web Appendix E: Comparison to Bayesian methods **11**

Web Appendix F: Multivariate t-distributed observations **13**

Web Appendix G: Contaminated Gaussian observations **17**

Web Appendix H: Multivariate log-Normal distributed observations **21**

Web Appendix I: Quality of estimation **25**

Web Appendix J: Reproducibility **30**

Web Appendix A: Empirical Bayes

We use empirical Bayes and estimate δ by the value maximizing the marginal (or integrated) likelihood of the model, i.e. by

$$\widehat{\delta} = \arg\max_{\delta} \, p(Y;\delta),$$

where

$$p(Y;\delta) = \pi^{-(np)/2} \frac{\Gamma_p\left(\frac{\delta+n}{2}\right)}{\Gamma_p\left(\frac{\delta}{2}\right)} \frac{\mid (\delta-p-1)\, D \mid^{\frac{\delta}{2}}}{\mid (\delta-p-1)\, D + S \mid^{\frac{\delta+n}{2}}}.$$

The above optimization problem is easily solved because the marginal likelihood is concave in δ. Moreover,

$$\mid (\delta-p-1)\, D \mid = (\delta-p-1)^p \prod_{r=1}^{R} d_r$$

and

$$\mid (\delta-p-1)\, D + S \mid = \left(\prod_{r=1}^{R} d_r\right)\left(\prod_{l=1}^{L} (\delta-p-1+e_l)\right),$$

where d_r and e_l are respectively the rth and lth largest eigenvalues of D and $D^{-1}S$. Hence, evaluating the objective function for different values of δ is computationally cheap provided the eigenvalues of D and $D^{-1}S$ have been pre-computed.

Web Appendix B: Bayes factor for marginal independence

Closed-form Bayes factor

We now derive an analytic expression for the Bayes factor evaluating the null hypothesis of marginal independence between any two variables in context of model (1). We test $\mathrm{H}^{\mathrm{M}}_{0,ij}: \ \sigma_{ij} = 0$ against the alternative hypothesis $\mathrm{H}^{\mathrm{M}}_{1,ij}: \sigma_{ij} \neq 0$, where σ_{ij} is the $(i,j)^{\text{th}}$ element of $\mathbf{\Sigma}$. The Bayes factor evaluating evidence in favour of $\mathrm{H}^{\mathrm{M}}_{1,ij}$ is

$$\mathrm{BF}^{\mathrm{M}}_{ij} = \frac{\int p_1(\mathbf{Y} \mid \mathbf{\Sigma}) p_1(\mathbf{\Sigma}) d\mathbf{\Sigma}}{\int p_0(\mathbf{Y} \mid \mathbf{\Sigma}^0) p_0(\mathbf{\Sigma}^0) d\mathbf{\Sigma}^0}, \tag{1}$$

where now $\mathbf{\Sigma}^0$ is such that $\sigma_{ij} = 0$.

We adopt a similar approach as in Section 3.1 to obtain (1) in closed-form. We first write the joint likelihood as $p(\mathbf{Y} \mid \boldsymbol{\Sigma}) = p(\mathbf{Y}_a \mid \boldsymbol{\Sigma}_{aa})p(\mathbf{Y}_b \mid \mathbf{Y}_a, \mathbf{B}_{b|a}, \boldsymbol{\Sigma}_{bb.a})$, and make a change of variable from $(\boldsymbol{\Sigma}_{aa}, \boldsymbol{\Sigma}_{ab}, \boldsymbol{\Sigma}_{bb})$ to $(\boldsymbol{\Sigma}_{aa}, \mathbf{B}_{b|a}, \boldsymbol{\Sigma}_{bb.a})$, where $\mathbf{B}_{b|a} = \boldsymbol{\Sigma}_{aa}^{-1}\boldsymbol{\Sigma}_{ab}$ and $\boldsymbol{\Sigma}_{bb.a} = \boldsymbol{\Sigma}_{bb} - \boldsymbol{\Sigma}_{ba}\boldsymbol{\Sigma}_{aa}^{-1}\boldsymbol{\Sigma}_{ab}$. Then, using the fact that $(\mathbf{B}_{b|a}, \boldsymbol{\Sigma}_{bb.a})$ is independent of $\boldsymbol{\Sigma}_{aa}$ it is easily seen that the Bayes factor (1) simplifies to

$$\mathrm{BF}_{ij}^{\mathrm{M}} = \frac{\int p_1(\mathbf{Y}_a \mid \boldsymbol{\Sigma}_{aa})p_1(\boldsymbol{\Sigma}_{aa})d\boldsymbol{\Sigma}_{aa}}{\int p_0(\mathbf{Y}_a \mid \boldsymbol{\Sigma}_{aa}^0)p_0(\boldsymbol{\Sigma}_{aa}^0)d\boldsymbol{\Sigma}_{aa}^0}. \tag{2}$$

Here, the densities under the alternative model, by properties of the multivariate normal and Inverse-Wishart distributions, are

$$\begin{aligned} \mathrm{vec}(\mathbf{Y}_a) \mid \boldsymbol{\Sigma}_{aa} &\sim N_{n\times 2}(0, \boldsymbol{\Sigma}_{aa} \otimes \mathbf{I}_n), \\ \boldsymbol{\Sigma}_{aa} &\sim IW_2(\mathbf{F}_{aa}, \delta - p + 2), \end{aligned} \tag{3}$$

whereas the densities under the null model are

$$\begin{aligned} \mathrm{vec}(\mathbf{Y}_a) \mid \boldsymbol{\Sigma}_{aa}^0 &\sim N_{n\times 2}(0, \boldsymbol{\Sigma}_{aa}^0 \otimes \mathbf{I}_n), \\ p_0(\boldsymbol{\Sigma}_{aa}^0) = p_1(\boldsymbol{\Sigma}_{aa} \mid \mathrm{H}_{0,ij}^{\mathrm{M}}) &= \frac{p_1(\boldsymbol{\Sigma}_{aa}, \mathrm{H}_{0,ij}^{\mathrm{M}})}{\int p_1(\boldsymbol{\Sigma}_{aa}, \mathrm{H}_{0,ij}^{\mathrm{M}})d\boldsymbol{\Sigma}_{aa}}. \end{aligned} \tag{4}$$

We now state the following lemma.

Lemma 3. *Assume (2) holds with densities defined by (3) and (4). Then the Bayes factor in favour of $H_{1,ij}^M$ is*

$$BF_{ij}^M = k_2(\delta, n, p)\frac{\left(1 - r_{f_{ij}}^2\right)^{\frac{\delta-p+2}{2}}}{\left(1 - r_{t_{ij}}^2\right)^{\frac{\delta+n-p+2}{2}}} \left(\frac{t_{ii}t_{jj}}{f_{ii}f_{jj}}\right)^{\frac{1}{2}},$$

with $k_2(\delta, n, p) = \dfrac{\Gamma_2\left(\frac{\delta+n-p+2}{2}\right)\Gamma^2\left(\frac{\delta-p+3}{2}\right)}{\Gamma_2\left(\frac{\delta-p+2}{2}\right)\Gamma^2\left(\frac{\delta+n-p+3}{2}\right)}$, $r_{f_{ij}} = \dfrac{f_{ij}}{\sqrt{f_{ii}f_{jj}}}$, $r_{t_{ij}} = \dfrac{t_{ij}}{\sqrt{t_{ii}t_{jj}}}$, $\mathbf{F}_{aa} = \begin{bmatrix} f_{ii} & f_{ij} \\ f_{ij} & f_{jj} \end{bmatrix}$, $\mathbf{T}_{aa} = \begin{bmatrix} t_{ii} & t_{ij} \\ t_{ij} & t_{jj} \end{bmatrix}$.

Remark 5. *In Lemma 3, the quantities* f_{ii} *and* t_{ii} *(resp.* f_{jj} *and* t_{jj}*) can be thought of representing prior and posterior marginal variances for coordinate* i *(resp.* j*), whereas* $r_{f_{ij}}$ *and* $r_{t_{ij}}$ *can be thought of representing prior and posterior marginal correlations.*

Remark 6. *The computation of the Bayes factor in Lemma 3 for all pairs of variables* (i, j)*,* $1 \leq i < j \leq p$*, boils down to computing* $\mathbf{T}$*.*

Consistency

Lemma 4. *If the sample correlation matrix has a limit as* $n \to \infty$ *that is positive definite, then the Bayes factor* BF^{M}_{ij} *is consistent in selection.*

Inference by multiple testing

We propose to infer the marginal independence graphs by multiple testing of hypotheses using the Bayes factor introduced in the previous Section. Precisely, we propose to infer the edge set $E_B = \{(i, j) \mid \sigma_{ij} \neq 0\}$ of the bidirected graph $B = (V, E_B)$ by evaluating $\mathrm{H}^{\mathrm{M}}_{0,ij} : \sigma_{ij} = 0$ (absence of an edge) versus $\mathrm{H}^{\mathrm{M}}_{1,ij} : \sigma_{ij} \neq 0$ (presence of an edge) for $1 \leq i < j \leq p$.

Scaled Bayes factors

To infer either graph structure it is necessary to compare Bayes factors between all $p(p-1)/2$ pairs of variables. However, the Bayes factor defined in Lemma 3 is not scale-invariant (due to its last term) and, hence, not comparable between different pairs of variables. In light of this, we define a scaled version of the Bayes factors that can more appropriately rank edges of graph B. Corollary 2 summarizes.

Corollary 2. *The scaled Bayes factor in favour of $H^M_{1,ij}$ is*

$$sBF^M_{ij} = k_2(\delta, n, p) \frac{\left(1 - r^2_{f_{ij}}\right)^{\frac{\delta-p+2}{2}}}{\left(1 - r^2_{t_{ij}}\right)^{\frac{\delta+n-p+2}{2}}},$$

with quantities defined as in Lemma 3.

Tail probability

We observe that the only term of the Bayes factor for marginal independence (defined in Corollary 2) that depends on the data is

$$r_{t_{ij}} = \frac{(f_{ii}f_{jj})^{1/2} r_{f_{ij}} + (s_{ii}s_{jj})^{1/2} r_{s_{ij}}}{(f_{ii} + s_{ii})^{1/2}(f_{jj} + s_{jj})^{1/2}},$$

via the elements of $\mathbf{S}_{aa} = \mathbf{Y}_a^T \mathbf{Y}_a = \{s_{ij}\}$. Here $r_{s_{ij}} = s_{ij}(s_{ii}s_{jj})^{-1/2}$. This means that we can write $pr\left\{\mathrm{sBF}^{\mathrm{M}}_{ij} > b_1\right\} = pr\left\{r^2_{s_{ij}} > c_1\right\}$, where c_1 is a quantity that depends on $\{\delta, n, f_{ii}, f_{jj}, r_{f_{ij}}, s_{ii}, s_{jj}\}$. Now, according to our model in equation (3) it is easily verified that $\mathbf{S}_{aa} \sim W_2(\mathbf{\Sigma}_{aa}, n)$ and, using Proposition 4, we can establish that $r^2_{s_{ij}} \mid \mathrm{H}^{\mathrm{M}}_{0,ij} \sim Beta(1/2, (n{-}1)/2)$. The tail probability of the Bayes factor can therefore be computed exactly using $Beta(1/2, (n{-}1)/2)$. We remark that the definition of the type I error is here conditioning on $\{\delta, n, f_{ii}, f_{jj}, r_{f_{ij}}, s_{ii}, s_{jj}\}$.

Web Appendix C: Tail probabilities

Proposition 3. *Suppose* $\mathbf{\Phi} \sim W_2(\mathbf{\Sigma}, d)$, *where*

$$\mathbf{\Phi} = \begin{pmatrix} \phi_1^2 & \phi_1\phi_2\varphi \\ \phi_1\phi_2\varphi & \phi_2^2 \end{pmatrix} \quad \text{and} \quad \mathbf{\Sigma} = \begin{pmatrix} \sigma_1^2 & \sigma_1\sigma_2\rho \\ \sigma_1\sigma_2\rho & \sigma_2^2 \end{pmatrix}$$

are parametrised in terms of their correlations $-1 \leq \varphi \leq 1$ *and* $-1 \leq \rho \leq 1$. *Then,*

$$(\varphi^2 \mid \rho = 0) \sim Beta(1/2, (d-1)/2).$$

Proposition 4. *The following equality holds:*

$$\mathbf{Y}_a^T\mathbf{Y}_a - \bar{\mathbf{B}}_{a|b}^T(\mathbf{Y}_b^T\mathbf{Y}_b + \mathbf{F}_{bb})\bar{\mathbf{B}}_{a|b} + \mathbf{F}_{ab}\mathbf{F}_{bb}^{-1}\mathbf{F}_{ba} = (\mathbf{Y}_a - \mathbf{Y}_b\mathbf{F}_{a|b})^T(\mathbf{I}_n + \mathbf{Y}_b\mathbf{F}_{bb}^{-1}\mathbf{Y}_b^T)^{-1}(\mathbf{Y}_a - \mathbf{Y}_b\mathbf{F}_{a|b}),$$

where $\bar{\mathbf{B}}_{a|b} = (\mathbf{Y}_b^T\mathbf{Y}_b + \mathbf{F}_{bb})^{-1}(\mathbf{Y}_b^T\mathbf{Y}_a + \mathbf{F}_{ba})$.

Proposition 5. *Let* $\mathbf{\Sigma}_{aa.b}$ *be fixed. Then, according to model (6) we have*

$$(\mathbf{Y}_a - \mathbf{Y}_b\mathbf{F}_{a|b})^T(\mathbf{I}_n + \mathbf{Y}_b\mathbf{F}_{bb}^{-1}\mathbf{Y}_b^T)^{-1}(\mathbf{Y}_a - \mathbf{Y}_b\mathbf{F}_{a|b}) \sim W_2(\mathbf{\Sigma}_{aa.b}, n).$$

Web Appendix D: Proofs

Proof of Proposition 1

Let $\alpha_\delta = (\delta - p - 1)/(\delta + n - p - 1) \in (0,1)$ depends on δ with n and p fixed. From the fact that $\widehat{\Sigma}_\delta = \alpha_\delta T + (1-\alpha_\delta)\widehat{\Sigma}_{\text{mle}}$, $\lim_{\delta\to\infty}\alpha_\delta = 1$ and $\lim_{\delta\to p+1}\alpha_\delta = 0$, it follows immediately that $\lim_{\delta\to\infty}\widehat{\Sigma}_\delta = T$ and $\lim_{\delta\to p+1}\widehat{\Sigma}_\delta = \widehat{\Sigma}_{\text{mle}}$, which prove (i) and (iii). Now, rewriting $\widehat{\Omega}_\delta = \frac{\delta+n}{\delta+n-p-1}\widehat{\Sigma}_\delta^{-1}$, it also becomes clear that $\lim_{\delta\to\infty}\widehat{\Omega}_\delta = T^{-1}$ and that $\lim_{\delta\to p+1}\widehat{\Omega}_\delta = \{(n+p+1)/n\}\,\widehat{\Sigma}_{\text{mle}}^{-1}$ (when $\widehat{\Sigma}_{\text{mle}}$ is positive definite), which prove (ii) and (iv). Finally, by definition $x^TTx > 0$ and $x^T\widehat{\Sigma}_{\text{mle}}x \geq 0$, $\forall x \in \mathbb{R}^p$, therefore we have $x^T\widehat{\Sigma}_\delta x = \alpha_\delta x^TTx + (1-\alpha_\delta)x^T\widehat{\Sigma}_{\text{mle}}x > 0$ and $\widehat{\Sigma}_\delta$ is positive definite for $0 < \alpha_\delta \leq 1$ or equivalently $\delta > 0$. As a direct consequence, $\widehat{\Omega}_\delta = \frac{\delta+n}{\delta+n-p-1}\widehat{\Sigma}_\delta^{-1}$ is also positive definite.

Proof of Proposition 2

Let $\alpha_n = (\delta - p - 1)/(\delta + n - p - 1) \in (0, 1)$ depends on n with δ and p fixed. From the fact that $\widehat{\Sigma}_\delta = \alpha_n T + (1 - \alpha_n)\widehat{\Sigma}_{\text{mle}}$ and that $\lim_{n\to\infty} \alpha_n = 0$, it follows immediately that $\lim_{n\to\infty} \widehat{\Sigma}_n = \widehat{\Sigma}_{\text{mle}}$. Rewriting $\widehat{\Omega}_n = \frac{\delta+n}{\delta+n-p-1}\widehat{\Sigma}_\delta^{-1}$ it is also found that $\lim_{n\to\infty} \widehat{\Omega}_n = \widehat{\Sigma}^{-1}_{\text{mle}}$.

Proof of Lemma 1

The numerator of the Bayes factor is

$$\iint p_1(Y_a \mid Y_b, B_{a|b}, \Sigma_{aa.b}) p_1(B_{a|b}, \Sigma_{aa.b}) dB_{a|b} d\Sigma_{aa.b} = \frac{|F_{bb}||F_{aa.b}|^{\frac{\delta}{2}}\Gamma_2\left(\frac{\delta+n}{2}\right)}{\pi^n \Gamma_2\left(\frac{\delta}{2}\right)|T_{bb}||T_{aa.b}|^{\frac{\delta+n}{2}}} . \tag{5}$$

Under $\mathrm{H}^{\mathrm{C}}_{0,ij}$, the model likelihood is

$$p_0(Y_a|Y_b, B_{a|b}, \Sigma^0_{aa.b}) = (2\pi)^{-n}(\omega_{ii}\omega_{jj})^{\frac{n}{2}} \exp\left\{-\frac{1}{2}\omega_{ii}(Y_a^{(i)} - Y_bB_{a|b}^{(i)})^T(Y_a^{(i)} - Y_bB_{a|b}^{(i)})\right\}$$
$$\times \exp\left\{-\frac{1}{2}\omega_{jj}(Y_a^{(j)} - Y_bB_{a|b}^{(j)})^T(Y_a^{(j)} - Y_bB_{a|b}^{(j)})\right\},$$

and the probability density of $(\Sigma^0_{aa.b}, B_{a|b})$ is

$$p_0(\Sigma^0_{aa.b}, B_{a|b}) = \frac{|F_{bb}|\left(g_{ii}g_{jj}\right)^{\frac{\delta+1}{2}}}{\pi^{(p-2)}2^{\delta+p-1}\Gamma^2\left(\frac{\delta+1}{2}\right)}(\omega_{ii}\omega_{jj})^{\frac{\delta+p+1}{2}} \exp\left\{-\frac{\omega_{ii}g_{ii} + \omega_{jj}g_{jj}}{2}\right\}$$
$$\times \exp\left\{-\frac{1}{2}\left(B^{(i)}_{a|b} - F^{(i)}_{a|b}\right)^T \omega_{ii}F_{bb}\left(B^{(i)}_{a|b} - F^{(i)}_{a|b}\right)\right\},$$
$$\times \exp\left\{-\frac{1}{2}\left(B^{(j)}_{a|b} - F^{(j)}_{a|b}\right)^T \omega_{jj}F_{bb}\left(B^{(j)}_{a|b} - F^{(j)}_{a|b}\right)\right\}$$

where $B^{(l)}_{a|b}$ and $Y^{(l)}_a$ represent the lth column of $B_{a|b}$ and Y_a. Therefore,

$$\iint p_0(Y_a|Y_b, B_{a|b}, \Sigma^0_{aa.b}) p_0(\Sigma^0_{aa.b}, B_{a|b}) dB_{a|b} d\Sigma^0_{aa.b} = \frac{|F_{bb}|\Gamma^2\left(\frac{\delta+n+1}{2}\right)\left(g_{ii}g_{jj}\right)^{\frac{\delta+1}{2}}}{\pi^n|T_{bb}|\Gamma^2\left(\frac{\delta+1}{2}\right)\left(q_{ii}q_{jj}\right)^{\frac{\delta+n+1}{2}}}. \tag{6}$$

Combining (5) and (6) we obtain the Bayes factor in Lemma 1.

Proof of Lemma 2

The numerator of the Bayes factor is

$$\int p_1(Y_a|\Sigma_{aa}) p_1(\Sigma_{aa}) d\Sigma_{aa} = \frac{|F_{aa}|^{\frac{\delta-p+2}{2}}\Gamma_2\left(\frac{\delta+n-p+2}{2}\right)}{\pi^n\Gamma_2\left(\frac{\delta-p+2}{2}\right)|T_{aa}|^{\frac{\delta+n-p+2}{2}}}. \tag{7}$$

Under $\mathrm{H}^{\mathrm{M}}_{0,ij}$, the model likelihood is

$$p_0(Y_a|\Sigma^0_{aa}) = (2\pi)^{-n}(\sigma_{ii}\sigma_{jj})^{-\frac{n}{2}} \exp\left\{-\frac{1}{2}\left(\sigma^{-1}_{ii}s_{ii} + \sigma^{-1}_{jj}s_{jj}\right)\right\},$$

and the probability density of Σ^0_{aa} is

$$p_0(\Sigma^0_{aa}) = \frac{(f_{ii}f_{jj})^{\frac{\delta-p+3}{2}}}{2^{\delta+1}\Gamma^2\left(\frac{\delta-p+3}{2}\right)}(\sigma_{ii}\sigma_{jj})^{-\frac{\delta-p+5}{2}} \exp\left\{-\frac{1}{2}\left(f_{ii}\sigma^{-1}_{ii} + f_{jj}\sigma^{-1}_{jj}\right)\right\},$$

As result,

$$\int p_0(Y_a|\Sigma^0_{aa})p_0(\Sigma^0_{aa})d\Sigma^0_{aa} = \frac{\Gamma^2\left(\frac{\delta+n-p+3}{2}\right)(f_{ii}f_{jj})^{\frac{\delta-p+3}{2}}}{\pi^n\Gamma^2\left(\frac{\delta-p+3}{2}\right)(t_{ii}t_{jj})^{\frac{\delta+n-p+3}{2}}}. \tag{8}$$

Combining (7) and (8) we obtain the Bayes factor in Lemma 2.

Proof of Lemma 3

Using Stirling's formula, the gamma function can asymptotically be approximated ($\approx$) by

$$\Gamma(\gamma_1 x + \gamma_2) \approx \sqrt{2\pi}\exp\{-\gamma_1 x\}(\gamma_1 x)^{\gamma_1 x+\gamma_2-1/2},$$

for large values of x (Wang and Maruyama, 2016). This means that for large values of n,

$$\Gamma_2\left\{(\delta+n-p+2)/2\right\} \approx 2\pi^{3/2}\exp(-n)(n/2)^{\frac{2n+2\delta-2p+1}{2}},$$

and

$$\Gamma^2\left\{(\delta+n-p+3)/2\right\} \approx 2\pi\exp(-n)(n/2)^{n+\delta-p+2}.$$

The Bayes factor in Lemma 2 is therefore asymptotically equivalent to

$$\mathrm{BF}^{\mathrm{M}}_{ij} \approx \frac{\Gamma_2\left(\frac{\delta+n-p+2}{2}\right)}{\Gamma^2\left(\frac{\delta+n-p+3}{2}\right)}\left(1-r^2_{s_{ij}}\right)^{-\frac{\delta+n-p+2}{2}} \approx \frac{(1-r^2_{s_{ij}})^{-\frac{\delta+n-p+2}{2}}}{n^{3/2}}$$

Now when $\mathrm{H}^{\mathrm{M}}_{0,ij}$ is true, $\lim_{n\to\infty} r^2_{s_{ij}} = 0$ because the sample correlation is asymptotically unbiased. Hence, $\lim_{n\to\infty}(1-r^2_{s_{ij}})^{-\frac{\delta+n-p+2}{2}} = 1$. Since $\lim_{n\to\infty} n^{3/2} = \infty$, we can conclude that $\lim_{n\to\infty} \mathrm{BF}^{\mathrm{M}}_{ij} = 0$, which proves the consistency under $\mathrm{H}^{\mathrm{M}}_{0,ij}$. On the other hand, when $\mathrm{H}^{\mathrm{M}}_{1,ij}$ is true, $\lim_{n\to\infty}(1-r^2_{s_{ij}})^{-1} = c_1$, $c_1 > 0$. The application of L'Hôpital's rule twice, by

deriving the numerator and denominator twice with respect to n, allows us to conclude that $\lim_{n\to\infty} \mathrm{BF}^{\mathrm{M}}_{ij} = \infty$. This completes our proof for the consistency of $\mathrm{BF}^{\mathrm{M}}_{ij}$.

Using similar arguments we prove the consistency of the Bayes factor in Lemma 1. The latter is asymptotically equivalent to

$$\mathrm{BF}^{\mathrm{C}}_{ij} \approx \frac{\Gamma\left(\frac{\delta+n}{2}\right)\Gamma\left(\frac{\delta+n-1}{2}\right)}{\Gamma^2\left(\frac{\delta+n+1}{2}\right)}(1-r^2_{q_{ij}})^{-\frac{\delta+n}{2}} \approx \frac{(1-r^2_{p_{ij}})^{-\frac{\delta+n}{2}}}{n^{3/2}},$$

where $r_{p_{ij}}$ denotes the sample partial correlation between variables i and j. On one hand, when $\mathrm{H}^{\mathrm{C}}_{0,ij}$ is true $\lim_{n\to\infty} r^2_{p_{ij}} = 0$ because the sample partial correlation is asymptotically unbiased. This implies that $\lim_{n\to\infty}(1-r^2_{p_{ij}})^{-\frac{\delta+n-p+2}{2}} = 1$. And because $\lim_{n\to\infty} n^{3/2} = \infty$ we can conclude that $\lim_{n\to\infty} \mathrm{BF}^{\mathrm{C}}_{ij} = 0$. On the other hand, when $\mathrm{H}^{\mathrm{C}}_{1,ij}$ is true, $\lim_{n\to\infty}(1-r^2_{p_{ij}})^{-1} = c_2$, $c_2 > 0$. Therefore, by applying L'Hôpital's rule twice, as above, it is found that $\lim_{n\to\infty} \mathrm{BF}^{\mathrm{C}}_{ij} = \infty$, which completes our proof for the consistency of $\mathrm{BF}^{\mathrm{C}}_{ij}$.

Proof of Proposition 3

This follows from the density of Φ. We have $p(\varphi \mid \rho = 0) \propto (1-\varphi^2)^{\frac{d-3}{2}}$ which implies that $p(\varphi^2 \mid \rho = 0) \propto \varphi^{-1}(1-\varphi^2)^{\frac{d-3}{2}}$.

Proof of Proposition 4

After some algebra we have

$$\begin{aligned} Y_a^T Y_a - \bar{B}^T_{a|b}(Y_b^T Y_b + F_{bb})\bar{B}_{a|b} + F_{ab}F_{bb}^{-1}F_{ba} = \\ Y_a^T\left\{I_n - Y_b\left(Y_b^T Y_b + F_{bb}\right)^{-1}Y_b^T\right\}Y_a \\ - 2Y_a^T Y_b\left(Y_b^T Y_b + F_{bb}\right)^{-1}F_{bb}F_{a|b} \\ + F^T_{a|b}\left\{F_{bb} - F_{bb}\left(Y_b^T Y_b + F_{bb}\right)^{-1}F_{bb}\right\}F_{a|b}, \end{aligned} \tag{9}$$

where, we recall, $\bar{B}_{a|b} = (Y_b^T Y_b + F_{bb})^{-1}(Y_b^T Y_a + F_{ba})$. Now,

$$Y_b\left(Y_b^T Y_b + F_{bb}\right)^{-1}F_{bb} = \left\{I_n - Y_b\left(Y_b^T Y_b + F_{bb}\right)^{-1}Y_b^T\right\}Y_b, \tag{10}$$

and, using twice the Sherman-Morrison-Woodbury matrix identity (Gupta and Nagar, 2000, Theorem 1.2.3.iv), it is found that

$$\begin{aligned} F_{bb} - F_{bb}\left(Y_b^T Y_b + F_{bb}\right)^{-1}F_{bb} &= \left\{F_{bb}^{-1} + (Y_b^T Y_b)^{-1}\right\}^{-1} \\ &= Y_b^T\left\{I_n - Y_b\left(Y_b^T Y_b + F_{bb}\right)^{-1}Y_b^T\right\}Y_b. \end{aligned} \tag{11}$$

Thus, by plugin in (10) and (11) in (9), we obtain

$$
\begin{aligned}
&Y_a^T Y_a - \bar{B}_{a|b}^T (Y_b^T Y_b + F_{bb}) \bar{B}_{a|b} + F_{ab} F_{bb}^{-1} F_{ba} = \\
&\left(Y_a - Y_b F_{a|b}\right)^T \left\{ I_n - Y_b \left(Y_b^T Y_b + F_{bb}\right)^{-1} Y_b^T \right\}^{-1} \left(Y_a - Y_b F_{a|b}\right),
\end{aligned}
$$

which is further reduced to

$$
\begin{aligned}
Y_a^T Y_a - &\bar{B}_{a|b}^T (Y_b^T Y_b + F_{bb}) \bar{B}_{a|b} + F_{ab} F_{bb}^{-1} F_{ba} = \\
&\left(Y_a - Y_b F_{a|b}\right)^T \left(I_n + Y_b F_{bb}^{-1} Y_b^T\right)^{-1} \left(Y_a - Y_b F_{a|b}\right),
\end{aligned}
$$

using, again, the Sherman-Morrison-Woodbury matrix identity.

Proof of Proposition 5

Consider $\Sigma_{aa.b}$ fixed in model (6). Then, $p(Y_a, B_{a|b} \mid Y_b, \Sigma_{aa.b}) \propto \exp\left\{-\frac{1}{2}\text{tr}\left[\Sigma_{aa.b}^{-1} C\right]\right\}$ where

$$
\begin{aligned}
C &= \left(Y_a - Y_b B_{a|b}\right)^T \left(Y_a - Y_b B_{a|b}\right) + \left(B_{a|b} - F_{a|b}\right)^T \left(B_{a|b} - F_{a|b}\right) \\
&= \left(B_{a|b} - \bar{B}_{a|b}\right)^T \left(B_{a|b} - \bar{B}_{a|b}\right) + Y_a^T Y_a - \bar{B}_{a|b}^T \left(Y_b^T Y_b + F_{bb}\right) \bar{B}_{a|b} + F_{ab} F_{bb}^{-1} F_{ba} \quad . \\
&= \left(B_{a|b} - \bar{B}_{a|b}\right)^T \left(B_{a|b} - \bar{B}_{a|b}\right) + \left(Y_a - Y_b F_{a|b}\right)^T \left(I_n + Y_b F_{bb}^{-1} Y_b^T\right)^{-1} \left(Y_a - Y_b F_{a|b}\right)
\end{aligned}
$$

Here the last equality is obtained using Proposition 4. As a result,

$$
\begin{aligned}
p(Y_a \mid Y_b, \Sigma_{aa.b}) &= \int p(Y_a, B_{a|b} \mid Y_b, \Sigma_{aa.b}) dB_{a|b} \\
&\propto \exp\left\{-\frac{1}{2}\text{tr}\left[\Sigma_{aa.b}^{-1} \left(Y_a - Y_b F_{a|b}\right)^T \left(I_n + Y_b F_{bb}^{-1} Y_b^T\right)^{-1} \left(Y_a - Y_b F_{a|b}\right)\right]\right\},
\end{aligned}
$$

which implies that $\text{vec}(Y_a) \mid Y_b, \Sigma_{aa.b} \sim N_{n\times 2}\left(\text{vec}(Y_b F_{a|b}), \Sigma_{aa.b} \otimes \left(I_n + Y_b F_{bb}^{-1} Y_b^T\right)\right)$.

Now, by the scaling property of the multivariate Gaussian distribution it is found that

$$
\text{vec}\left\{\left(I_n + Y_b F_{bb}^{-1} Y_b^T\right)^{-\frac{1}{2}} \left(Y_a - Y_b F_{a|b}\right)\right\} \mid \Sigma_{aa.b} \sim N_{n\times 2}\left(0, \Sigma_{aa.b} \otimes I_n\right),
$$

and it follows that (Gupta and Nagar, 2000, Theorem 3.2.2.)

$$
(Y_a - Y_b F_{a|b})^T (I_n + Y_b F_{bb}^{-1} Y_b^T)^{-1} (Y_a - Y_b F_{a|b}) \sim W_2(\Sigma_{aa.b}, n).
$$

Web Appendix E: Comparison to Bayesian methods

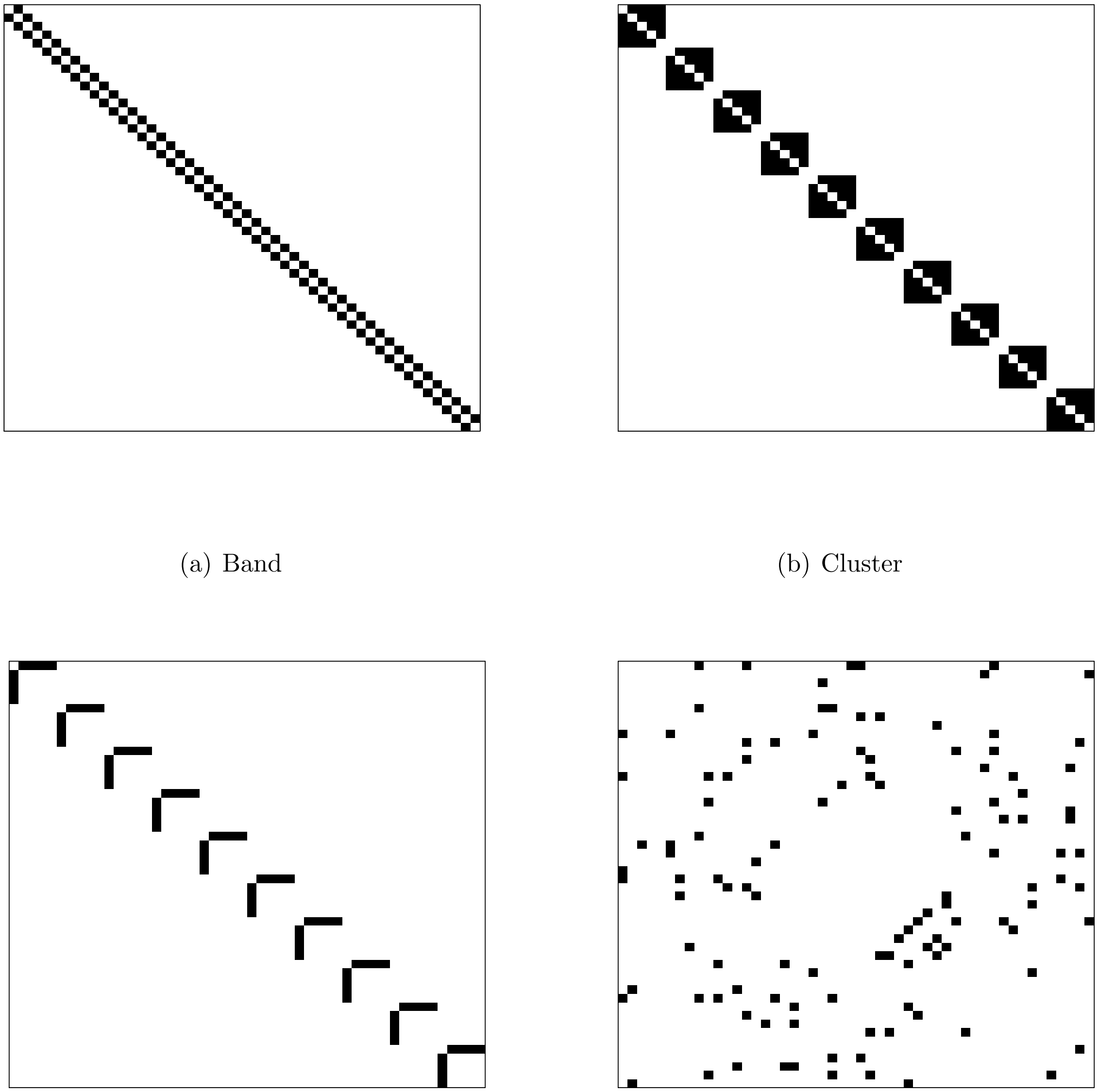

(a) Band (b) Cluster

(c) Hub (d) Random

Web Figure 1. *Graph structures considered in the simulation. Black and white dots represent non-zero and zero entries in* Φ*, respectively. Only off-diagonal elements are displayed. The graph density* η*, that is the ratio of the number of edges and the number of possible edges, is (a)* $\eta = 0.080$*, (b)* $\eta = 0.167$*, (c)* $\eta = 0.067$ *and (d)* $\eta = 0.083$*.*

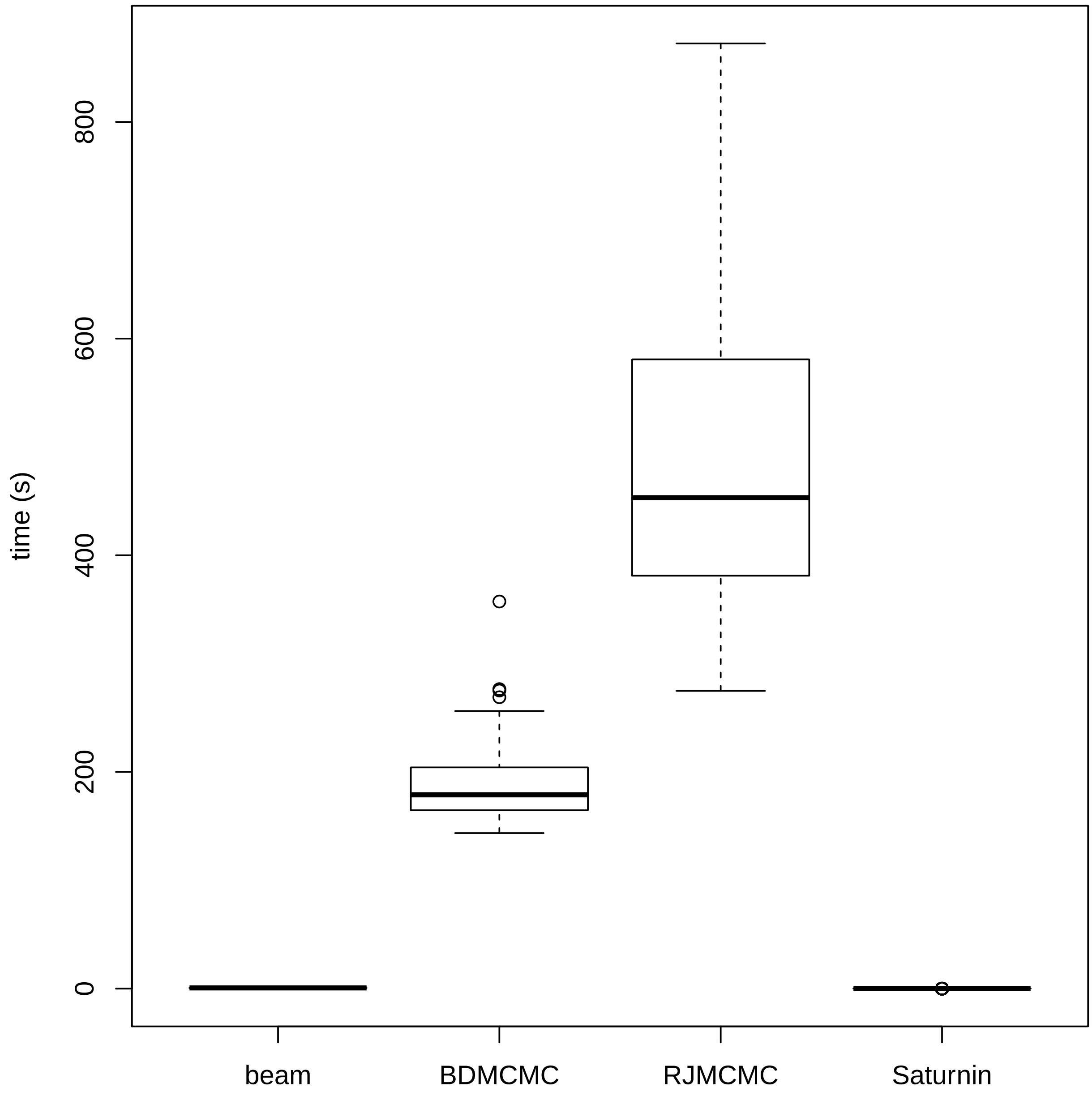

Web Figure 2. *Running time in seconds (assessed on 3.40GHz Intel Core i7-3770 CPU) for each Bayesian method under comparison.*

Web Appendix F: Multivariate t

Band structure

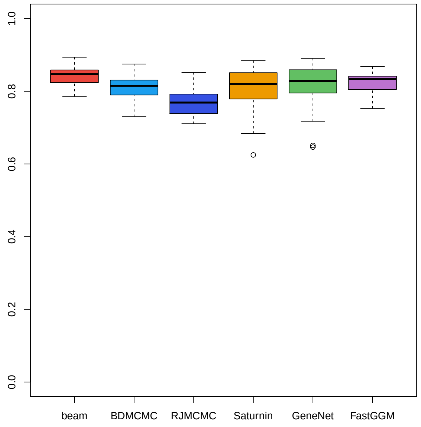

(a) ROC, $n = 100$

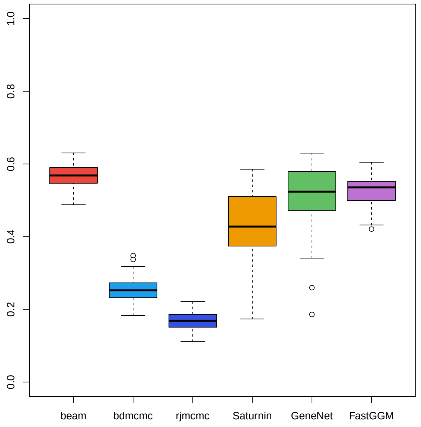

(b) PR, $n = 100$

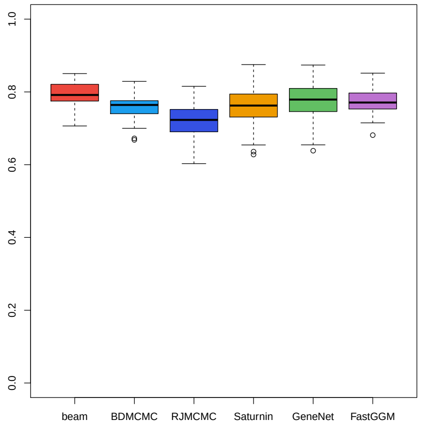

(c) ROC, $n = 50$

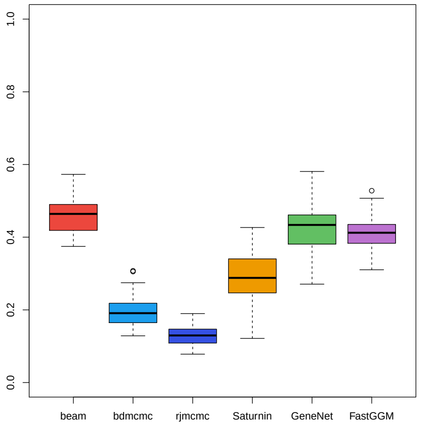

(d) PR, $n = 50$

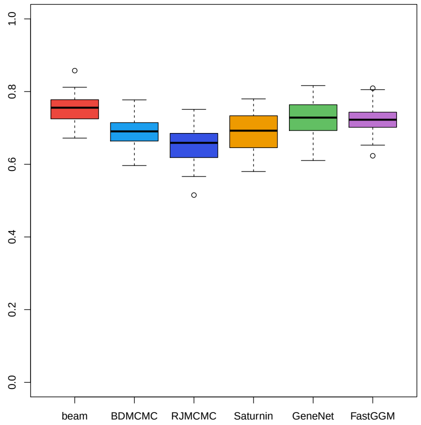

(e) ROC, $n = 25$

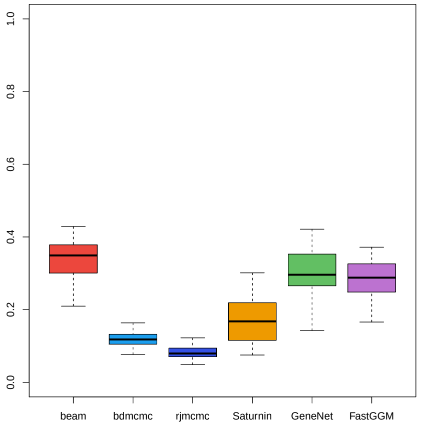

(f) PR, $n = 25$

Web Figure 3. *Areas under the receiver operating characteristic (ROC) and precision-recall (PR) curves for **scnenario 1** (multivariate t distribution) with **band structure**.*

Cluster structure

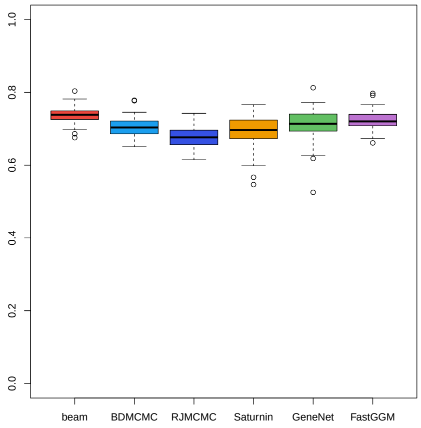

(a) ROC, $n = 100$

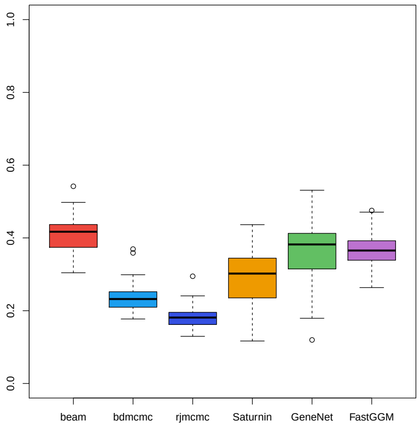

(b) PR, $n = 100$

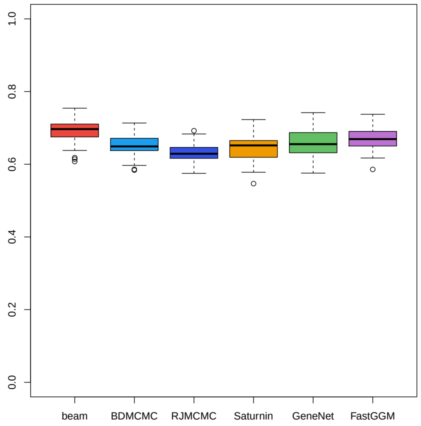

(c) ROC, $n = 50$

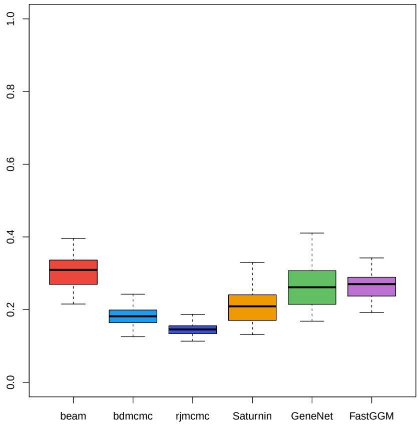

(d) PR, $n = 50$

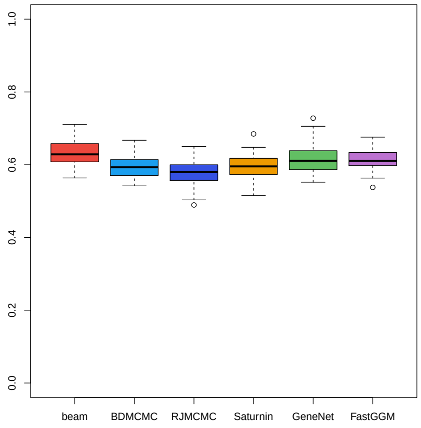

(e) ROC, $n = 25$

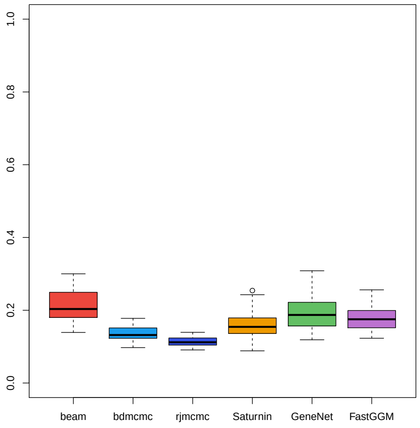

(f) PR, $n = 25$

Web Figure 4. *Areas under the receiver operating characteristic (ROC) and precision-recall (PR) curves for* ***scnenario 1*** *(multivariate t distribution) with* ***cluster structure****.*

Hub structure

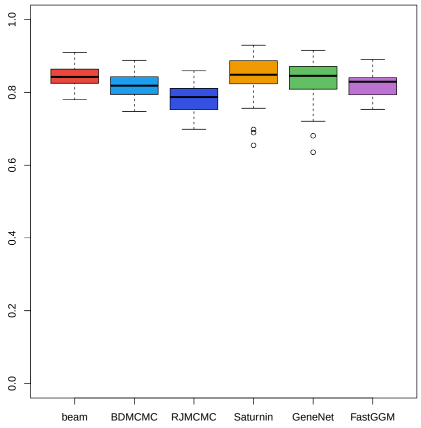

(a) ROC, $n = 100$

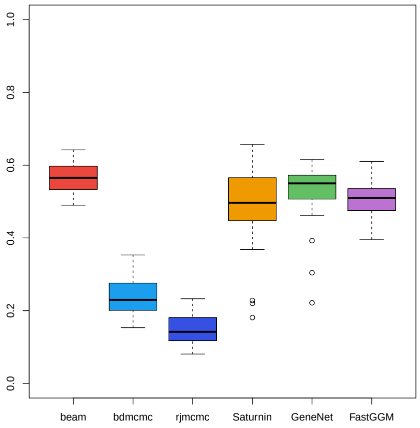

(b) PR, $n = 100$

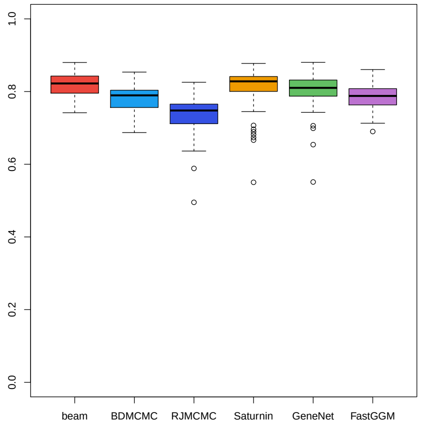

(c) ROC, $n = 50$

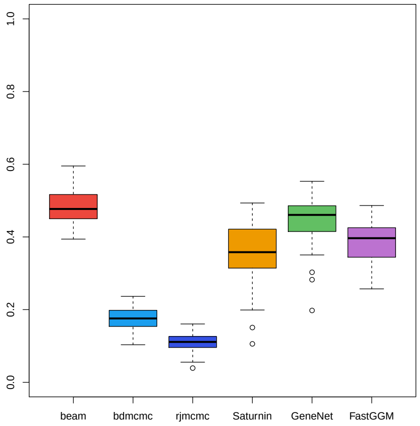

(d) PR, $n = 50$

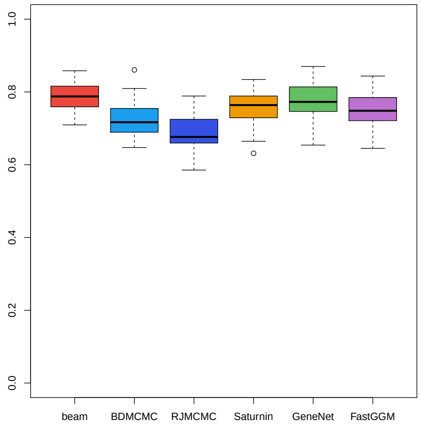

(e) ROC, $n = 25$

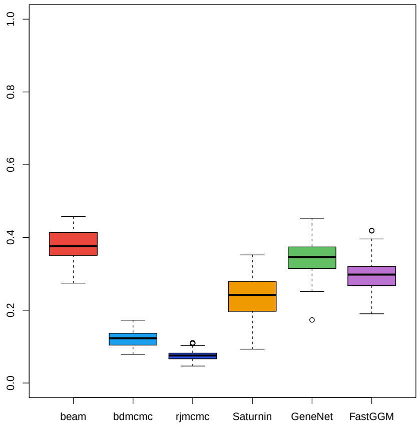

(f) PR, $n = 25$

Web Figure 5. *Areas under the receiver operating characteristic (ROC) and precision-recall (PR) curves for* ***scnenario 1*** *(multivariate t distribution) with* ***hub structure****.*

Random structure

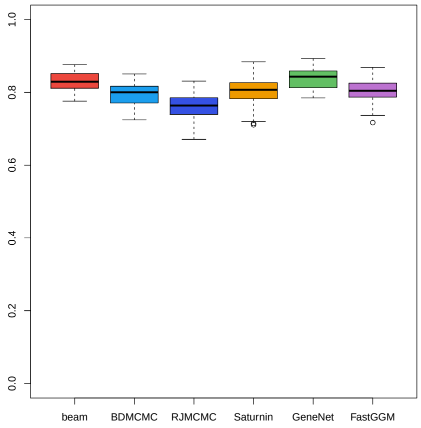

(a) ROC, $n = 100$

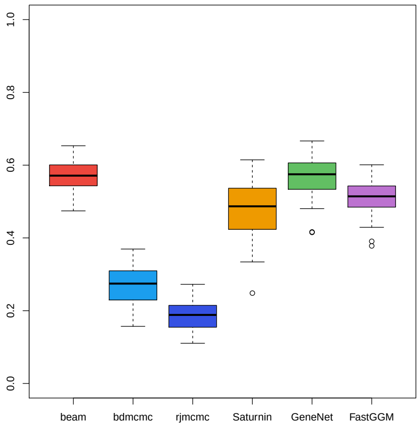

(b) PR, $n = 100$

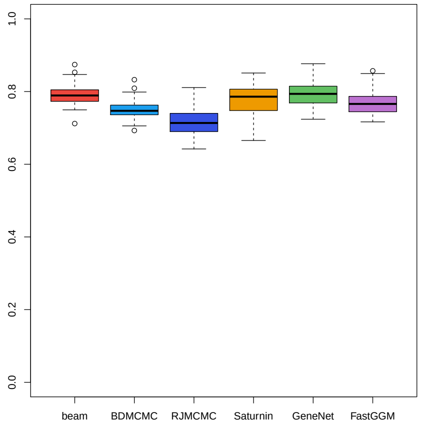

(c) ROC, $n = 50$

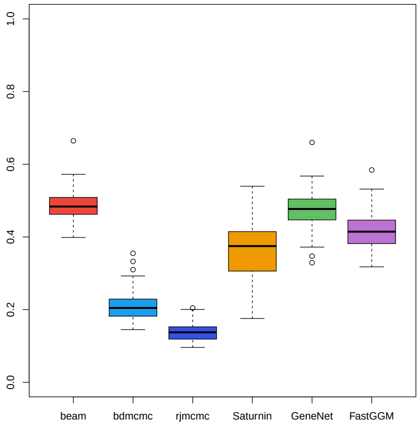

(d) PR, $n = 50$

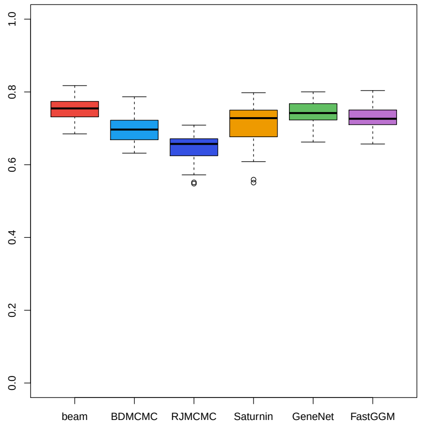

(e) ROC, $n = 25$

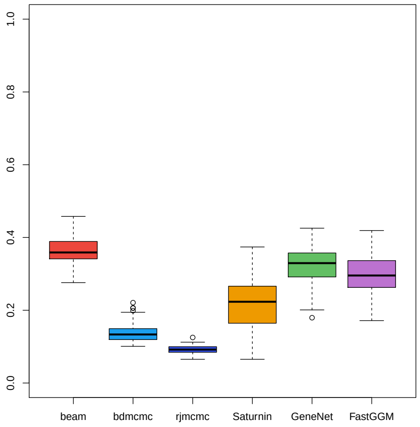

(f) PR, $n = 25$

Web Figure 6. *Areas under the receiver operating characteristic (ROC) and precision-recall (PR) curves for* ***scenario 1*** *(multivariate t distribution) with* ***random structure****.*

Web Appendix G: Contaminated Gaussian observations

Band structure

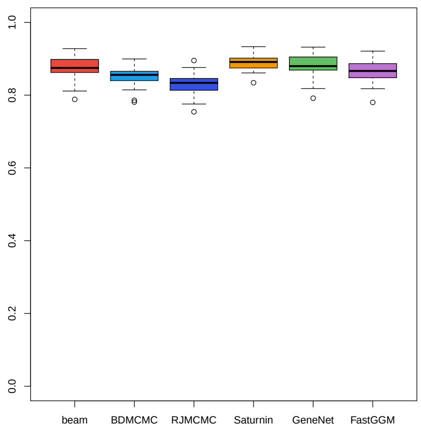

(a) ROC, $n = 100$

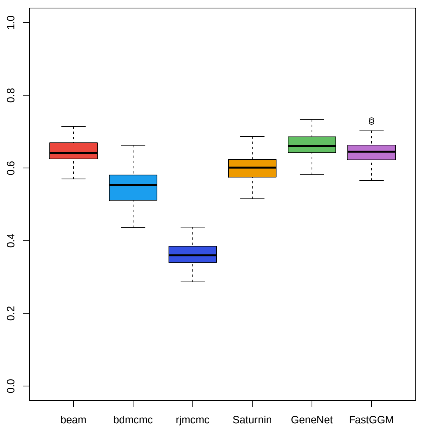

(b) PR, $n = 100$

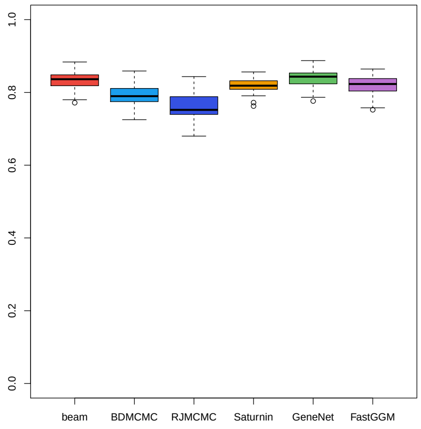

(c) ROC, $n = 50$

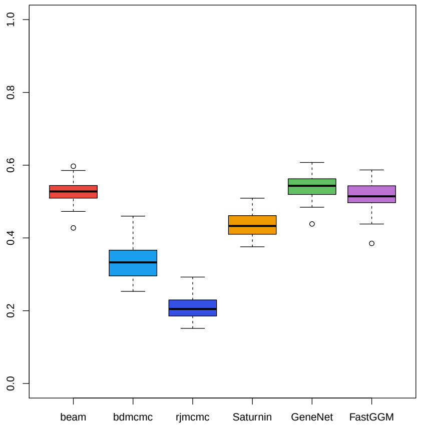

(d) PR, $n = 50$

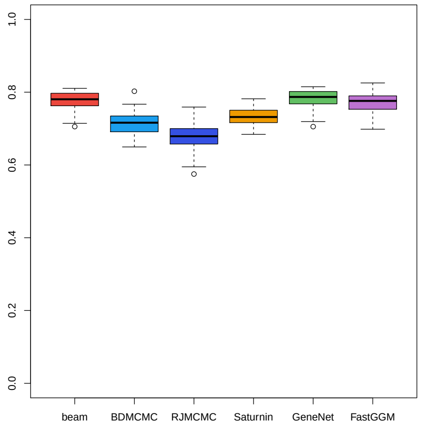

(e) ROC, $n = 25$

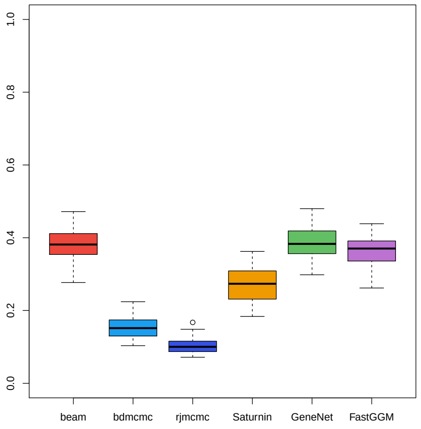

(f) PR, $n = 25$

Web Figure 7. *Areas under the receiver operating characteristic (ROC) and precision-recall (PR) curves for* ***scnenario 1*** *(multivariate t distribution) with* ***band structure****.*

Cluster structure

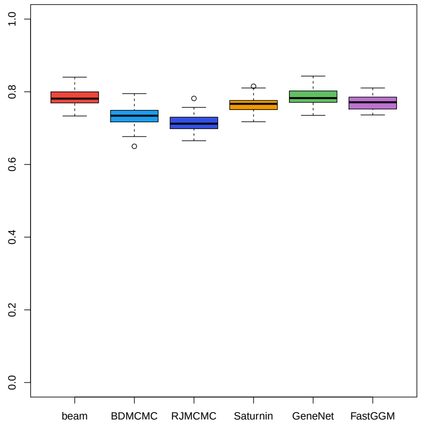

(a) ROC, $n = 100$

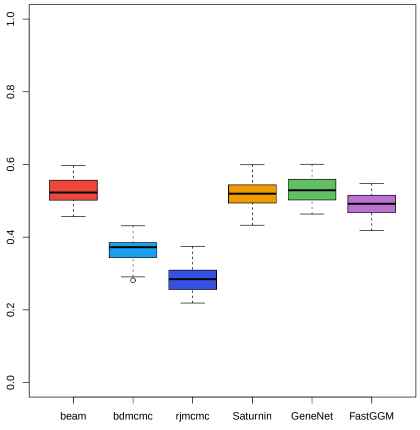

(b) PR, $n = 100$

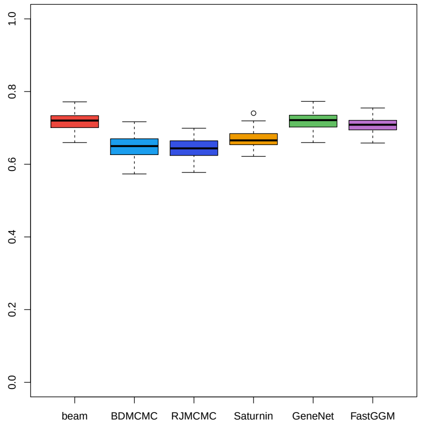

(c) ROC, $n = 50$

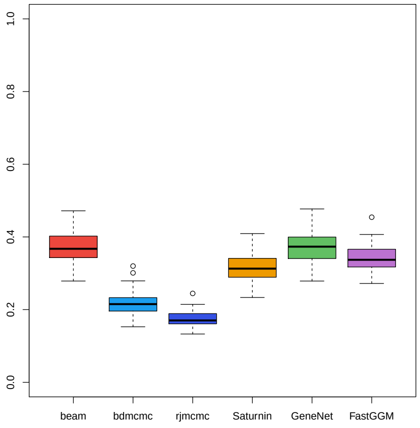

(d) PR, $n = 50$

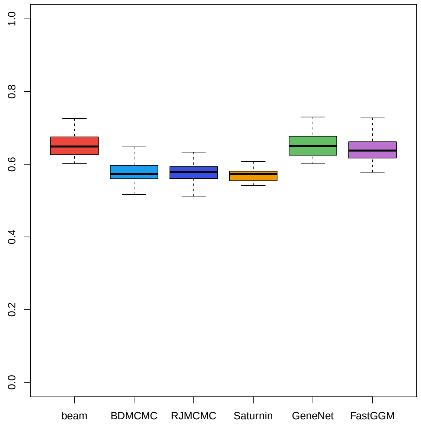

(e) ROC, $n = 25$

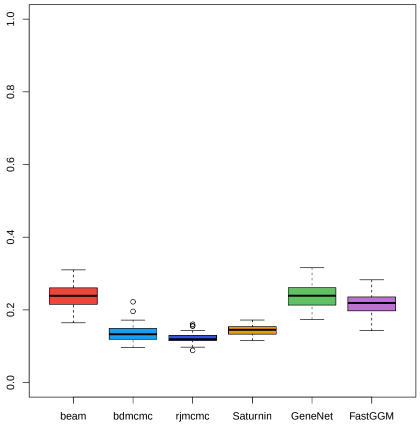

(f) PR, $n = 25$

Web Figure 8. *Areas under the receiver operating characteristic (ROC) and precision-recall (PR) curves for* ***scnenario 1*** *(multivariate t distribution) with* ***cluster structure****.*

Hub structure

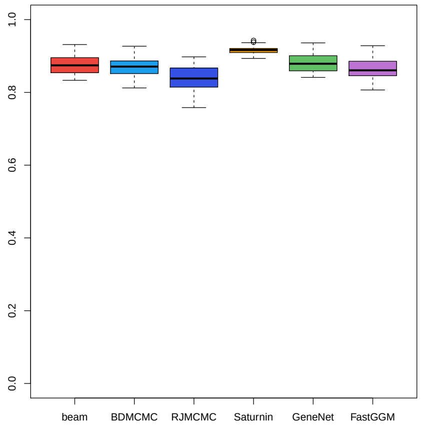

(a) ROC, $n = 100$

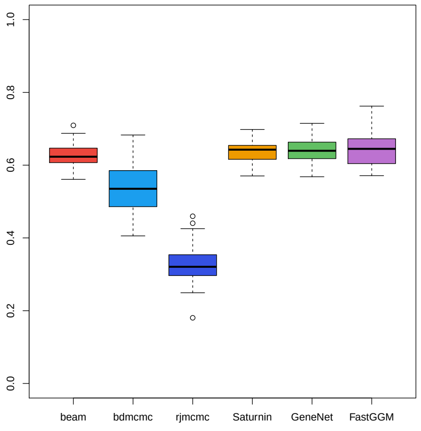

(b) PR, $n = 100$

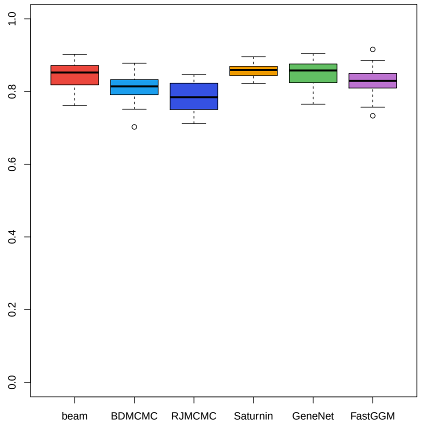

(c) ROC, $n = 50$

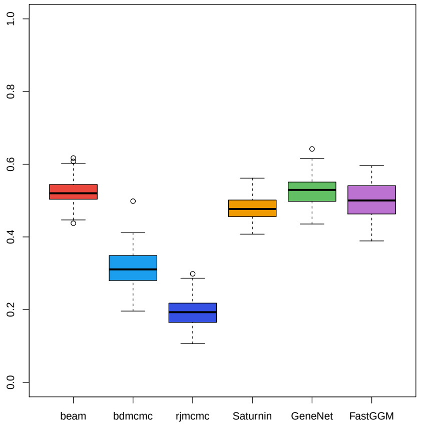

(d) PR, $n = 50$

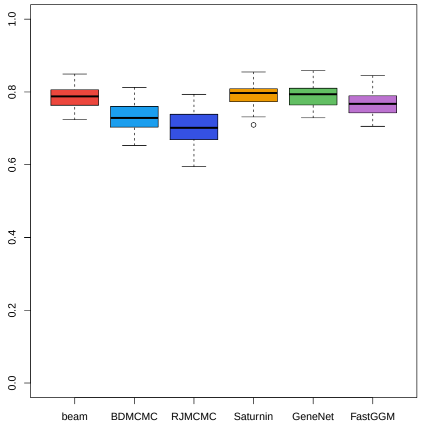

(e) ROC, $n = 25$

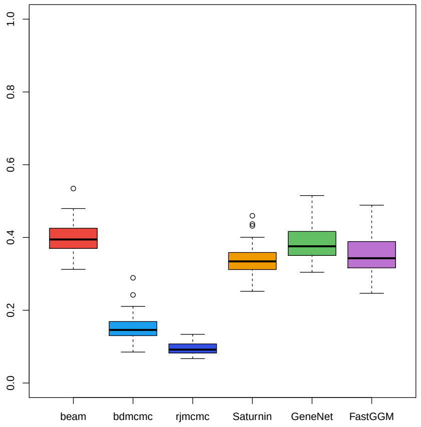

(f) PR, $n = 25$

Web Figure 9. *Areas under the receiver operating characteristic (ROC) and precision-recall (PR) curves for* ***scnenario 1*** *(multivariate t distribution) with* ***hub structure****.*

Random structure

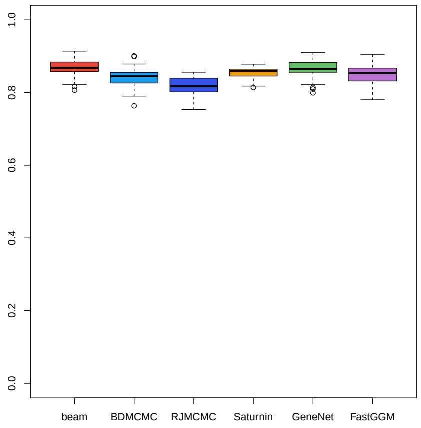

(a) ROC, $n = 100$

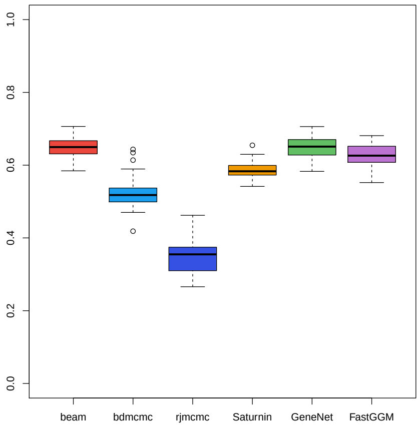

(b) PR, $n = 100$

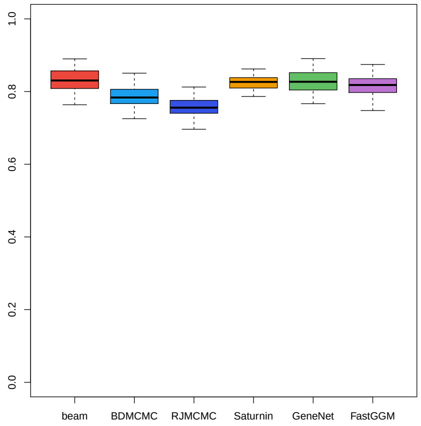

(c) ROC, $n = 50$

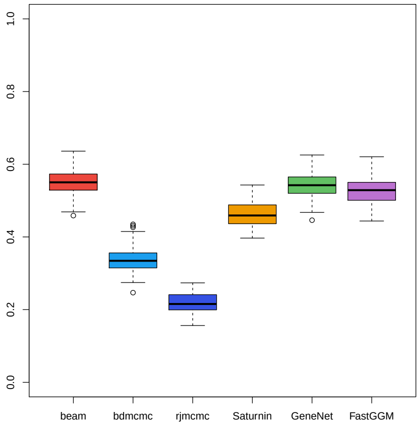

(d) PR, $n = 50$

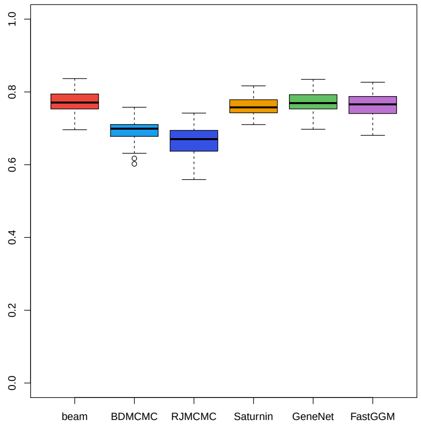

(e) ROC, $n = 25$

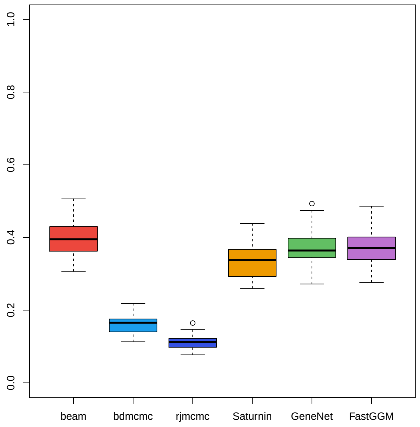

(f) PR, $n = 25$

Web Figure 10. *Areas under the receiver operating characteristic (ROC) and precision-recall (PR) curves for* ***scnenario 1*** *(multivariate t distribution) with* ***random structure****.*

Web Appendix H: Multivariate log-Normal

Band structure

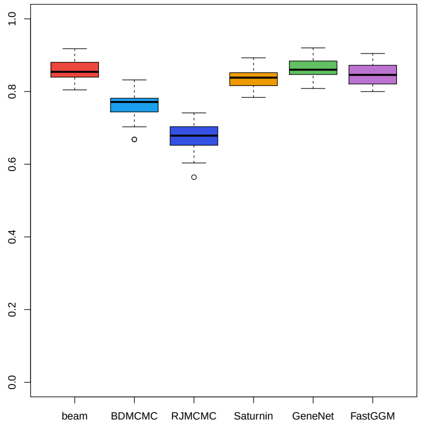

(a) ROC, $n = 100$

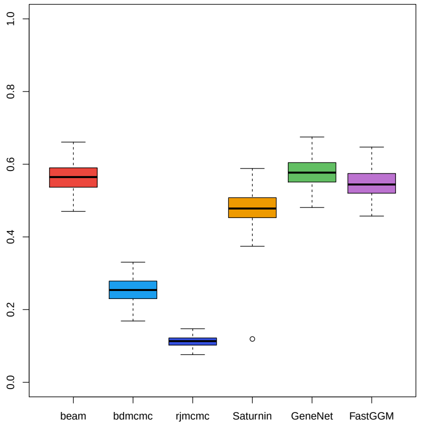

(b) PR, $n = 100$

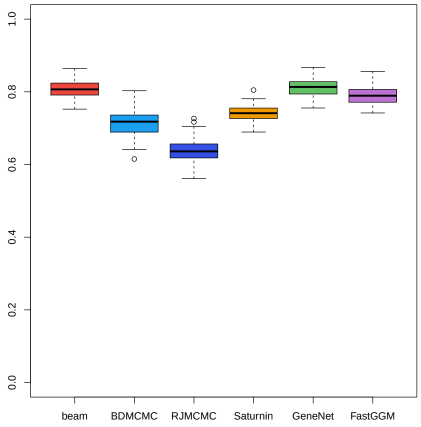

(c) ROC, $n = 50$

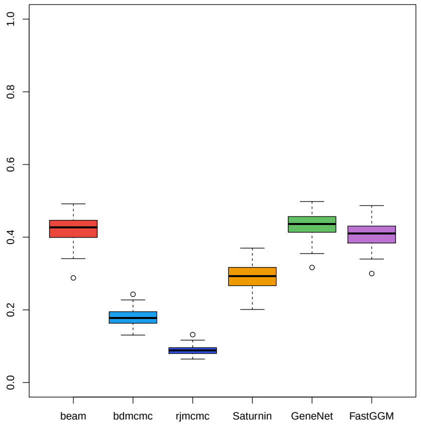

(d) PR, $n = 50$

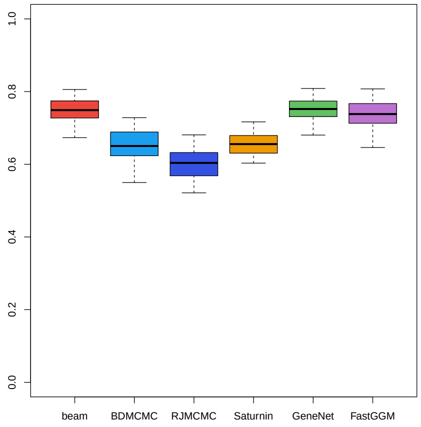

(e) ROC, $n = 25$

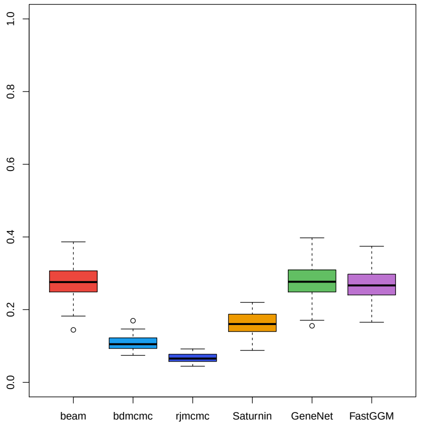

(f) PR, $n = 25$

Web Figure 11. *Areas under the receiver operating characteristic (ROC) and precision-recall (PR) curves for* ***scnenario 1*** *(multivariate t distribution) with* ***band structure****.*

Cluster structure

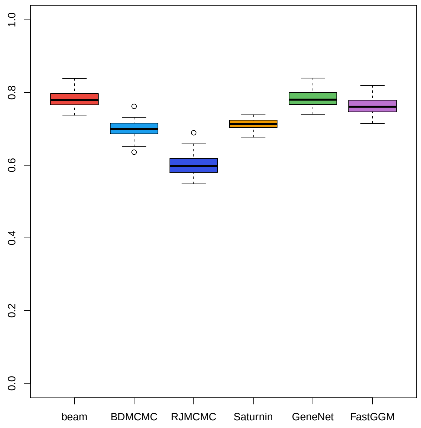

(a) ROC, $n = 100$

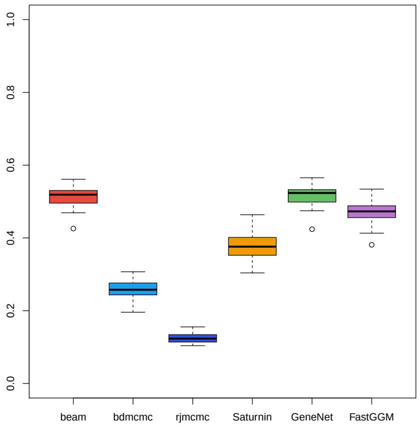

(b) PR, $n = 100$

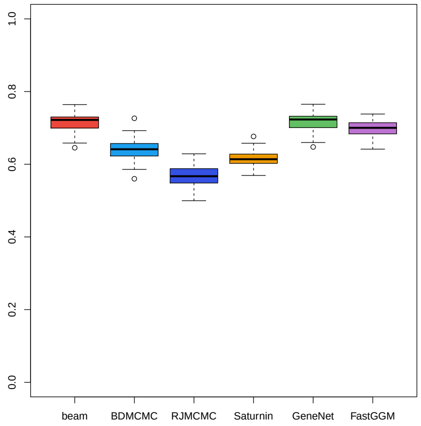

(c) ROC, $n = 50$

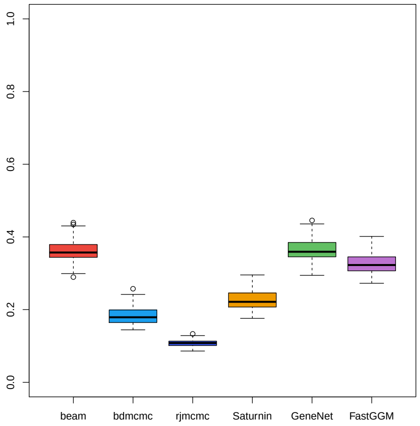

(d) PR, $n = 50$

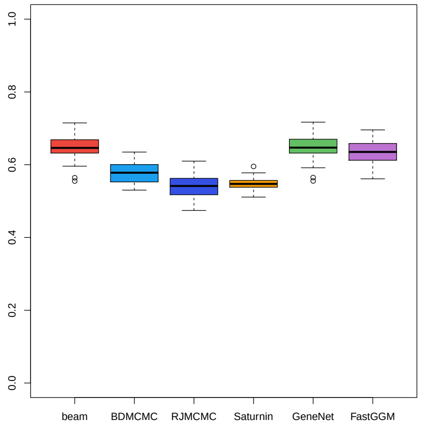

(e) ROC, $n = 25$

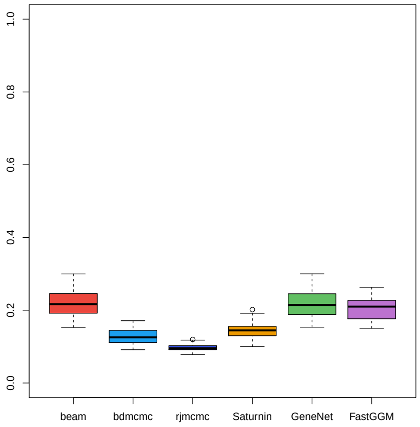

(f) PR, $n = 25$

Web Figure 12. *Areas under the receiver operating characteristic (ROC) and precision-recall (PR) curves for **scnenario 1** (multivariate t distribution) with **cluster structure**.*

Hub structure

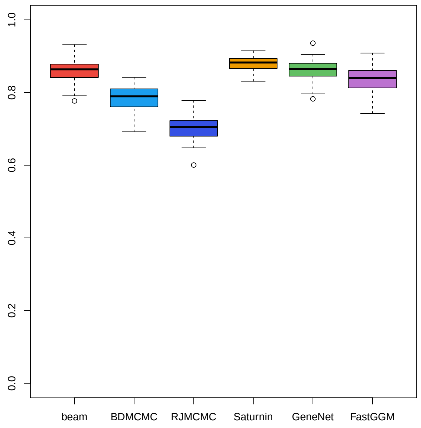

(a) ROC, $n = 100$

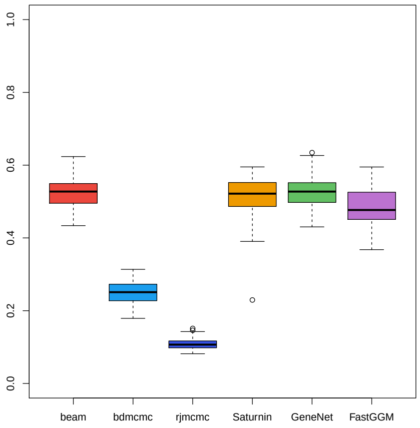

(b) PR, $n = 100$

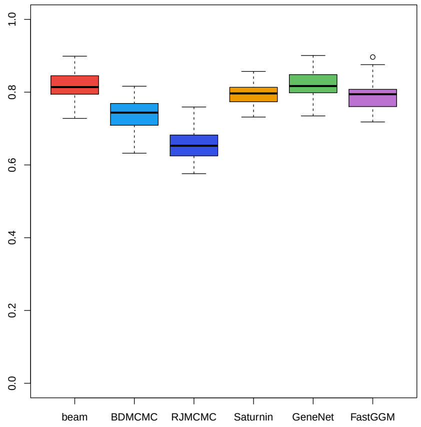

(c) ROC, $n = 50$

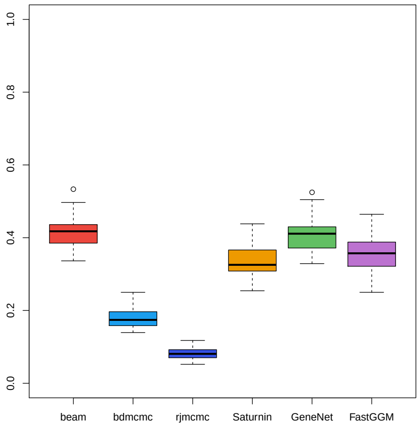

(d) PR, $n = 50$

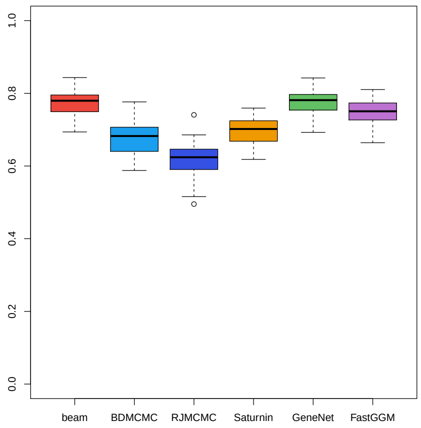

(e) ROC, $n = 25$

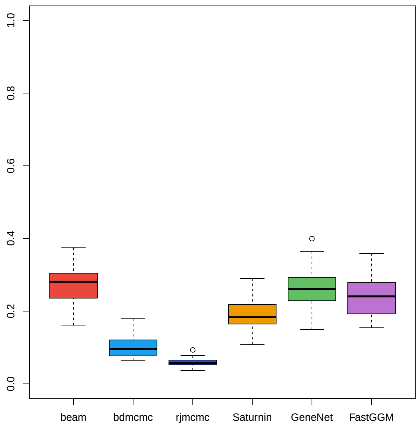

(f) PR, $n = 25$

Web Figure 13. *Areas under the receiver operating characteristic (ROC) and precision-recall (PR) curves for* ***scnenario 1*** *(multivariate t distribution) with* ***hub structure****.*

Random structure

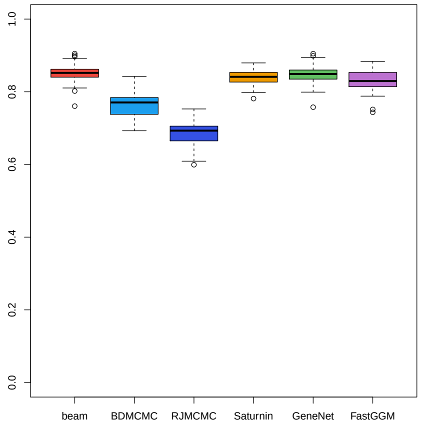

(a) ROC, $n = 100$

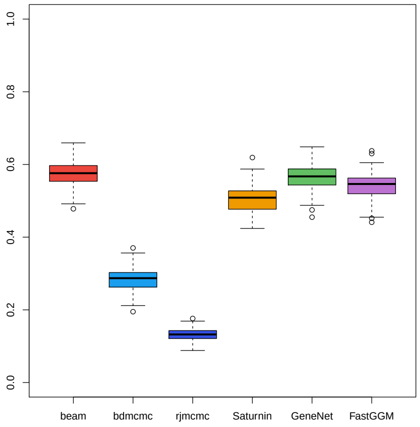

(b) PR, $n = 100$

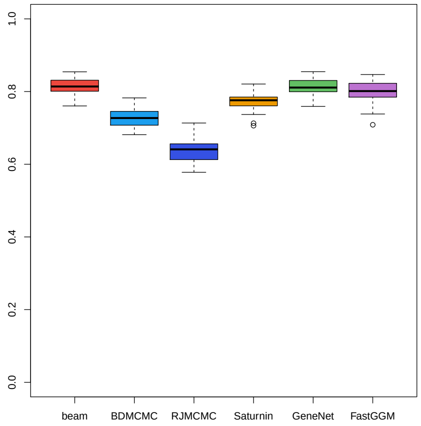

(c) ROC, $n = 50$

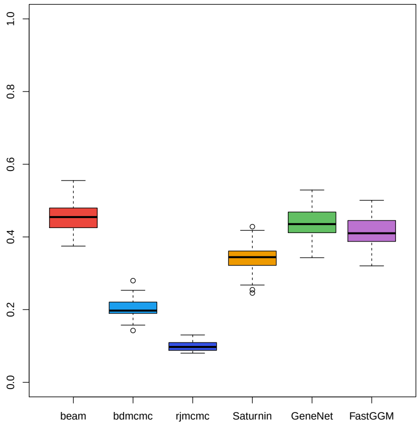

(d) PR, $n = 50$

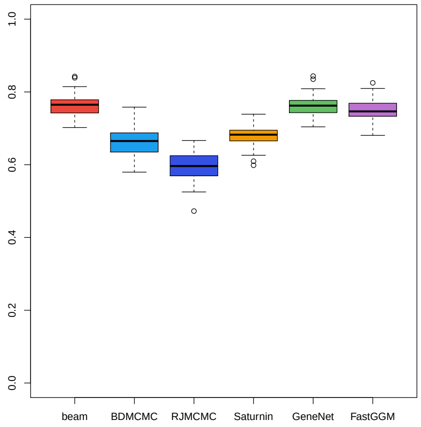

(e) ROC, $n = 25$

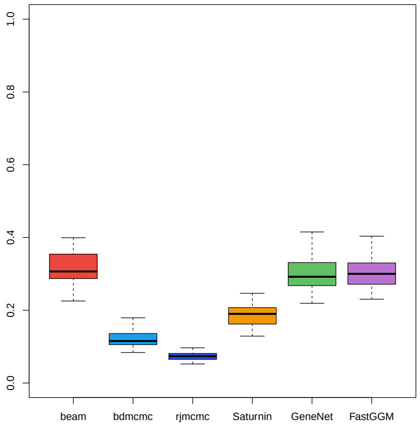

(f) PR, $n = 25$

Web Figure 14. *Areas under the receiver operating characteristic (ROC) and precision-recall (PR) curves for* ***scenario 1*** *(multivariate t distribution) with* ***random structure****.*

Web Appendix I: Quality of estimation

Setting

We assess the estimation accuracy of our method and that of Bayesian and non-Bayesian contenders considered in the paper. We additionally consider two versions of our method, when the shrinkage target D equals the true covariance matrix (beam++) and when the shrinkage target D equals the true covariance matrix where half of the rows and columns have been permuted (beam+). These two versions of our method aim to illustrate the effect of incorporating prior information, when this information is 100% and 50% true, respectively. We fix $p = 25$ and generate 50 datasets of size $n \in \{25, 50\}$ using the same four graphical structures considered in Section 5.1 of the paper.

Results

Results show that our method performs as good as GeneNet, better than FastGGM, but not as good as sampling-based Bayesian methods. This is expected because our method employs a linear shrinkage estimator whereas sampling-based Bayesian methods employ a sparse estimator that is more flexible. However, results show that our method outperforms sampling-based Bayesian methods when prior information is incorporated via the prior matrix D.

Band structure

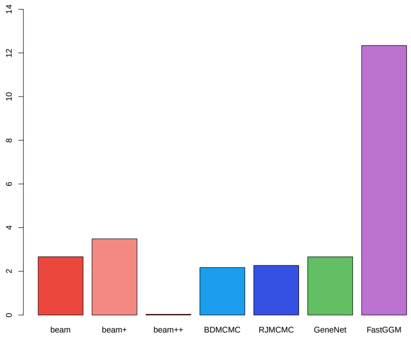

(a) all partial correlations, $n = 25$

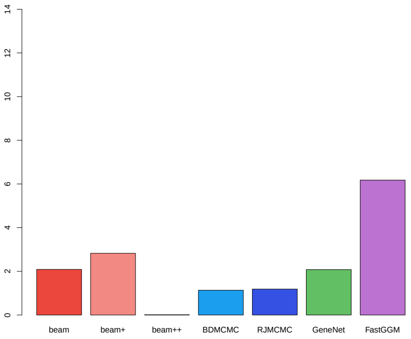

(b) all partial correlations, $n = 50$

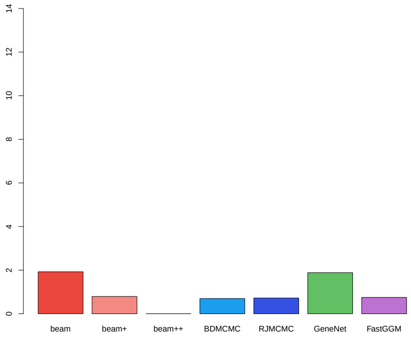

(c) non-zero partial correlations, $n = 25$

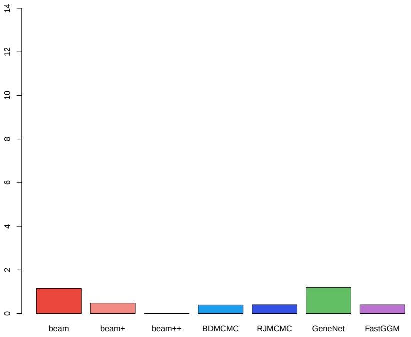

(d) non-zero partial correlations, $n = 50$

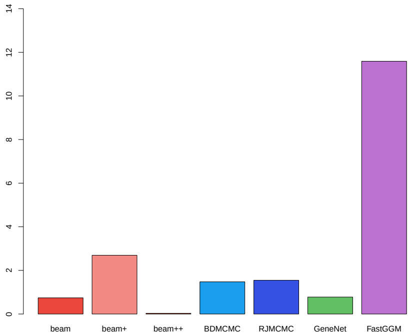

(e) zero partial correlations, $n = 25$

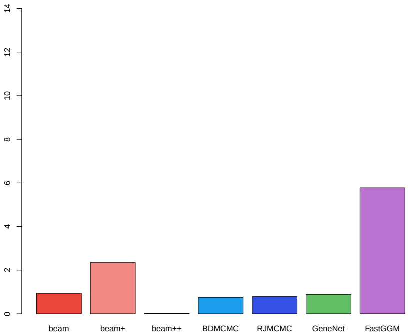

(f) zero partial correlations, $n = 50$

Web Figure 15. *Frobenius loss for all, non-zero and zero true partial correlations when the graph has a* ***band structure****.*

Cluster structure

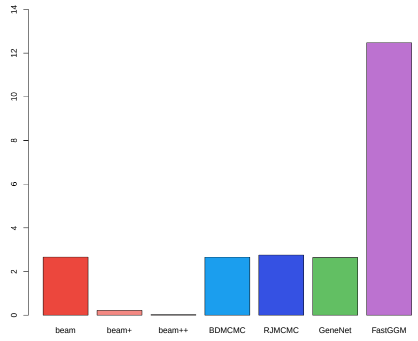

(a) all partial correlations, $n = 25$

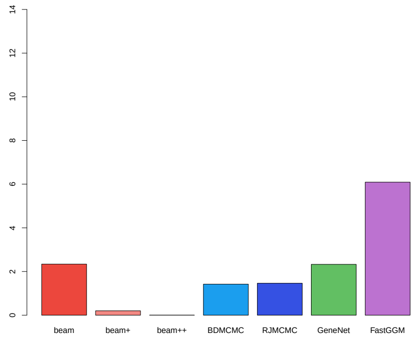

(b) all partial correlations, $n = 50$

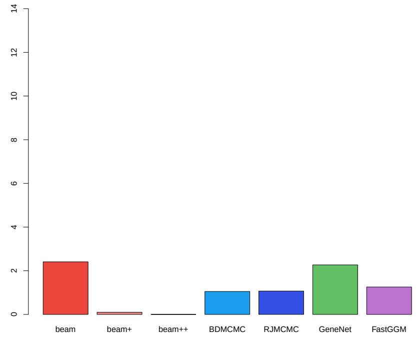

(c) non-zero partial correlations, $n = 25$

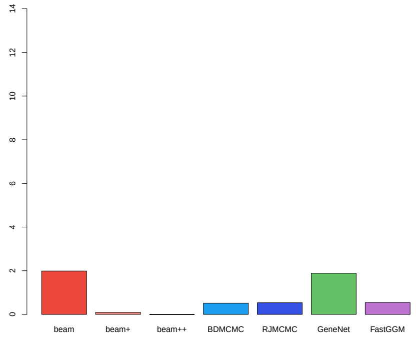

(d) non-zero partial correlations, $n = 50$

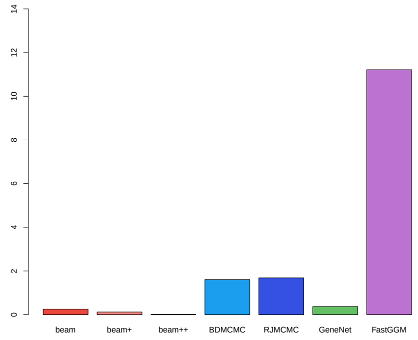

(e) zero partial correlations, $n = 25$

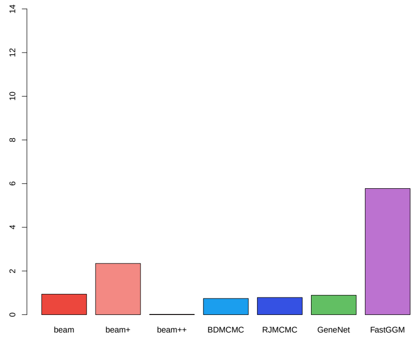

(f) zero partial correlations, $n = 50$

Web Figure 16. *Frobenius loss for all, non-zero and zero true partial correlations when the graph has a* ***cluster structure****.*

Hub structure

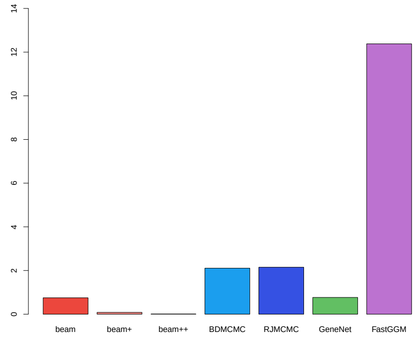

(a) all partial correlations, $n = 25$

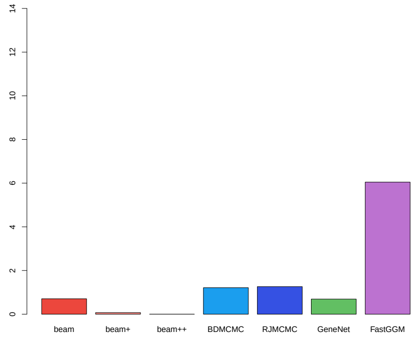

(b) all partial correlations, $n = 50$

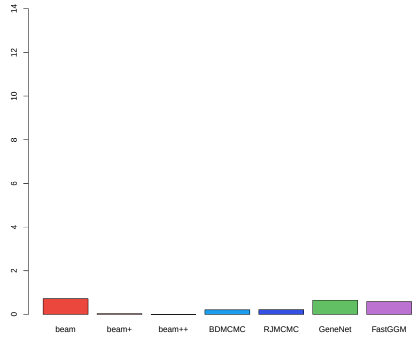

(c) non-zero partial correlations, $n = 25$

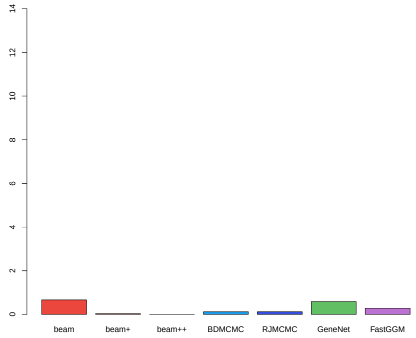

(d) non-zero partial correlations, $n = 50$

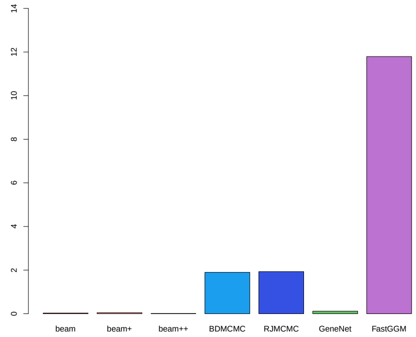

(e) zero partial correlations, $n = 25$

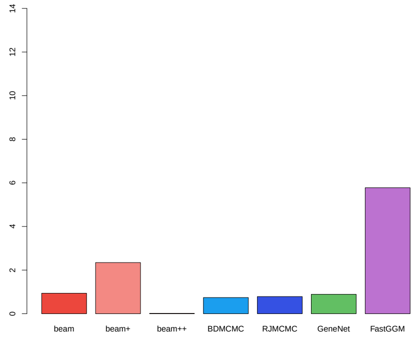

(f) zero partial correlations, $n = 50$

Web Figure 17. *Frobenius loss for all, non-zero and zero true partial correlations when the graph has a* ***hub structure****.*

Random structure

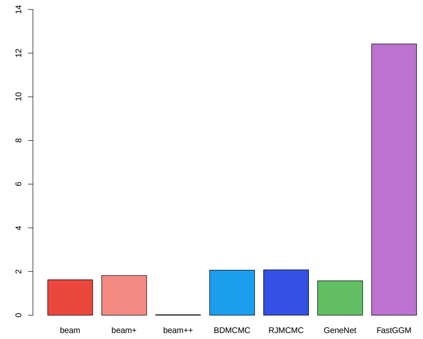

(a) all partial correlations, $n = 25$

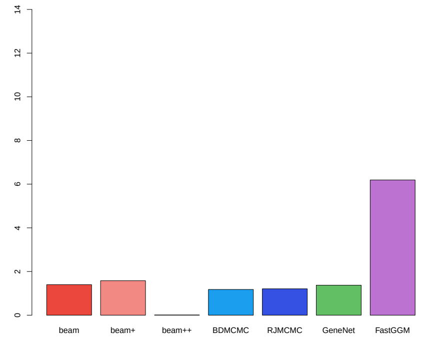

(b) all partial correlations, $n = 50$

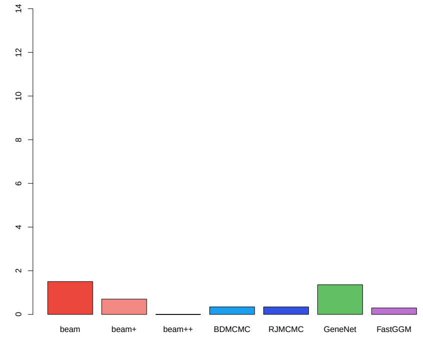

(c) non-zero partial correlations, $n = 25$

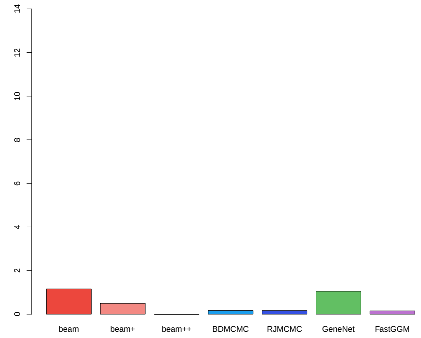

(d) non-zero partial correlations, $n = 50$

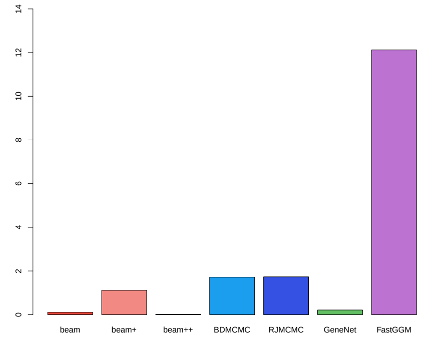

(e) zero partial correlations, $n = 25$

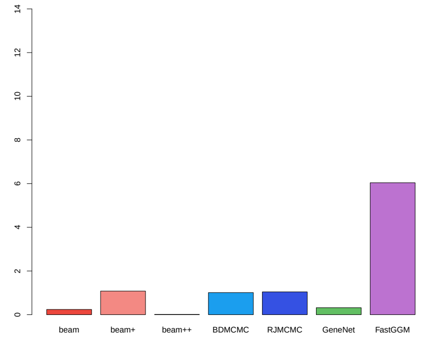

(f) zero partial correlations, $n = 50$

Web Figure 18. *Frobenius loss for all, non-zero and zero true partial correlations when the graph has a* ***random structure****.*

Web Appendix J: Reproducibility

We carry out a small data-based simulation to illustrate the reproducibility of the different methods on real data. We focused on the subset of the TCGA gene expression data that comprise the $k = 500$ most variable genes. Then, we randomly split 50 times the n by k data set into two equally sized data sets of size $n/2$. We applied our method `beam`, as well as contenders `saturnin`, `GeneNet` and `FastGGM` in each half over all 50 random splits. For computational reasons, sampling-based Bayesian methods were not included in the comparison. Figure 19 displays, for each method, the number of overlapping edges among the top 50 and 100 edges (over the 50 random splits) between the two halves. Results show that our method provides more reproducible edges than contenders on real data.

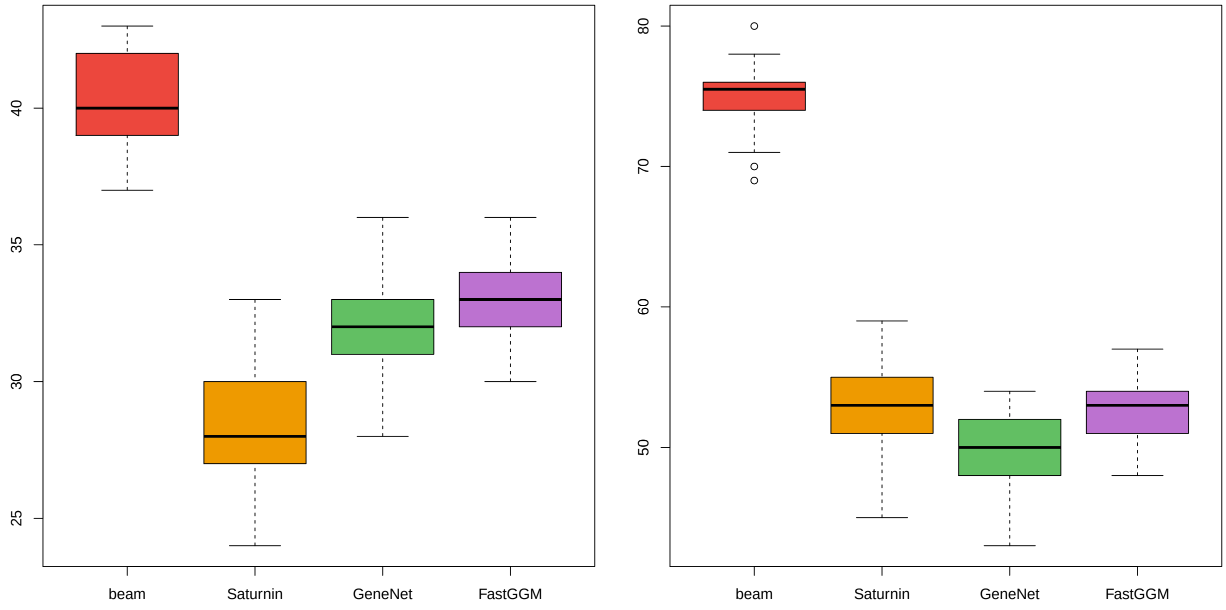

(a) (b)

Web Figure 19. *Number of overlapping edges in the top (a) 50 and (b) 100 strongest ones in two equally-sized splits of the TCGA gene expression data for* `beam`*,* `saturnin`*,* `GeneNet` *and* `FastGGM`*.*

References

Gupta, A. K. and Nagar, D. K. (2000). *Matrix variate distributions*, volume 104 of *Chapman & Hall/CRC Monographs and Surveys in Pure and Applied Mathematics*. Chapman & Hall/CRC, Boca Raton, FL. *Referred to on pages 9 and 10.*

Wang, M. and Maruyama, Y. (2016). Consistency of Bayes factor for nonnested model selection when the model dimension grows. *Bernoulli* **22,** 2080–2100. *Referred to on page 8.*
